# Chemoenzymatic Hunsdiecker-Type Decarboxylative Bromination of Cinnamic Acids

**DOI:** 10.1021/acscatal.2c00485

**Published:** 2022-04-04

**Authors:** Huanhuan Li, Sabry H. H. Younes, Shaohang Chen, Peigao Duan, Chengsen Cui, Ron Wever, Wuyuan Zhang, Frank Hollmann

**Affiliations:** † School of Chemical Engineering and Technology, Xi’an Jiaotong University, Xi’an 710049, China; ‡ Department of Biotechnology, Delft University of Technology, Van der Maasweg 9, Delft 2629HZ, The Netherlands; § Department of Chemistry, Faculty of Sciences, Sohag University, Sohag 82524, Egypt; ∥ Tianjin Institute of Industrial Biotechnology, Chinese Academy of Sciences, 32 West 7th Avenue, Tianjin 300308, China; ⊥ National Center of Technology Innovation for Synthetic Biology, 32 West 7th Avenue, Tianjin 300308, China; # Van’t Hoff Institute for Molecular Sciences, University of Amsterdam, Amsterdam 1098 XH, The Netherlands

**Keywords:** biocatalysis, Hunsdiecker reaction, decarboxylation, vinyl bromides, unsaturated carboxylic acids, vanadium chloroperoxidase

## Abstract

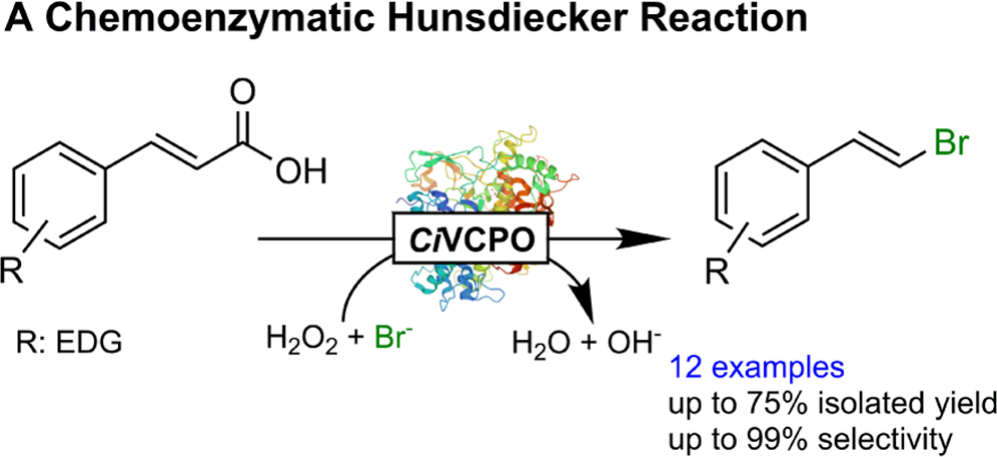

In this contribution, we report chemoenzymatic bromodecarboxylation (Hunsdiecker-type) of α,ß-unsaturated carboxylic acids. The extraordinarily robust chloroperoxidase from *Curvularia inaequalis* (*Ci*VCPO) generated hypobromite from H_2_O_2_ and bromide, which then spontaneously reacted with a broad range of unsaturated carboxylic acids and yielded the corresponding vinyl bromide products. Selectivity issues arising from the (here undesired) addition of water to the intermediate bromonium ion could be solved by reaction medium engineering. The vinyl bromides so obtained could be used as starting materials for a range of cross-coupling and pericyclic reactions.

## Introduction

Vinyl halides are versatile intermediates in organic chemistry, especially as starting materials in carbon–carbon cross-coupling reactions.^1−3^ Halodecarboxylation of α,β-unsaturated carboxylic acids represents a convenient synthetic access to a broad range of vinyl halides.^4^ In addition to the classical Hunsdiecker reaction^5^ starting from silver carboxylates and its later modifications such as the Cristol–Firth modification (utilizing HgO as a catalyst)^6^ and the Kochi reaction (utilizing stoichiometric amounts of Pb(OAc)_4_),^7^ some metal-free alternatives have been developed. The Barton reaction, for example, utilizes organic hypohalites as stoichiometric reagents,^8^ while the Suarez reaction is based on hypervalent iodosobenzene diacetates.^9^ More recently, *N*-halo succinimide (NXS)^4,10^ reagents have become dominant as a source for electrophilic halide species to initiate the halodecarboxylation reaction.

From an environmental and practical point of view, stoichiometric halide sources such as NXS^10^ or other *N*-halides^11^ may be questionable due to the formation of large amounts of succinimide waste products lowering the atom efficiency of the transformation and complicating product isolation and purification. Therefore, alternative methods for the *in situ* generation of electrophilic halides have been investigated comprising chemical^12,13^ or electrochemical halide oxidation^14^ methods. Particularly, vanadate^15−18^ and molybdate^19^ complexes have been investigated as mimetics for haloperoxidase enzymes. Their poor catalytic activity, however, necessitates high catalyst loadings of up to 10–50 mol %.

Already in 1985, Izumi and co-workers have pioneered an enzymatic approach for the oxidative generation of hypohalites with H_2_O_2_ and chloroperoxidase from *Caldariomyces fumago* (*Cf*CPO) as a biocatalyst.^20^ Unfortunately, these pioneering contributions have not resulted in great interest from the research community, which can largely be ascribed to the difficulties using *Cf*CPO as a catalyst.^21,22^ In addition to the issues in recombinant production of this catalyst, predominantly, it’s poor robustness against the stoichiometric oxidant (H_2_O_2_) represents a major practical hurdle.

**Scheme 1:**
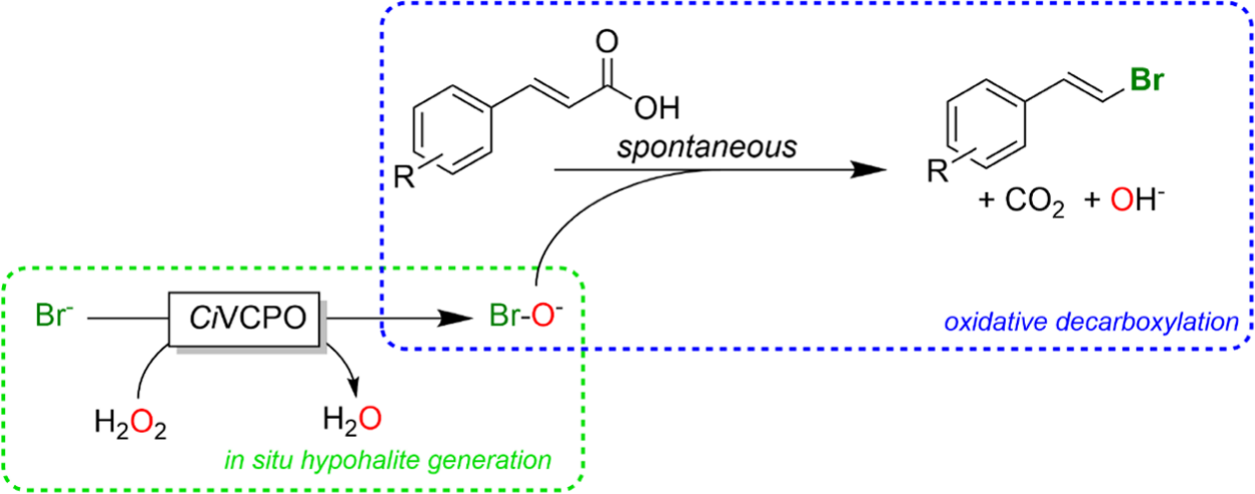
Envisioned Biocatalytic Hunsdiecker-Type Reaction The overall reaction comprises a biocatalytic step in which the reactive halide species (hypohalite) is formed *in situ* from halides and H_2_O_2_ catalyzed by the V-dependent chloroperoxidase from *C. inaequalis* (*Ci*VCPO). In the second step, the hypobromite spontaneously (nonenzyme-mediated) reacts with the starting material inducing the bromodecarboxylation reaction.

With this in mind, we set out to evaluate whether the vanadium-dependent chloroperoxidase from *Curvularia inaequalis* (*Ci*VCPO) may be a more suitable (bio)catalyst to promote H_2_O_2_-driven bromodecarboxylation reactions (Scheme 1). *Ci*VCPO^23−26^ excels as a robust and active enzyme tolerating high concentrations of H_2_O_2_ and organic solvents. Overall, a chemoenzymatic reaction scheme was envisioned wherein *Ci*VCPO catalyzes the H_2_O_2_-driven oxidation of bromide to hypobromite with the latter spontaneously (nonenzymatically) reacting with α,ß-unsaturated carboxylic acids yielding the corresponding vinyl bromide and CO_2_.

## Results and Discussion

**Figure 1:**
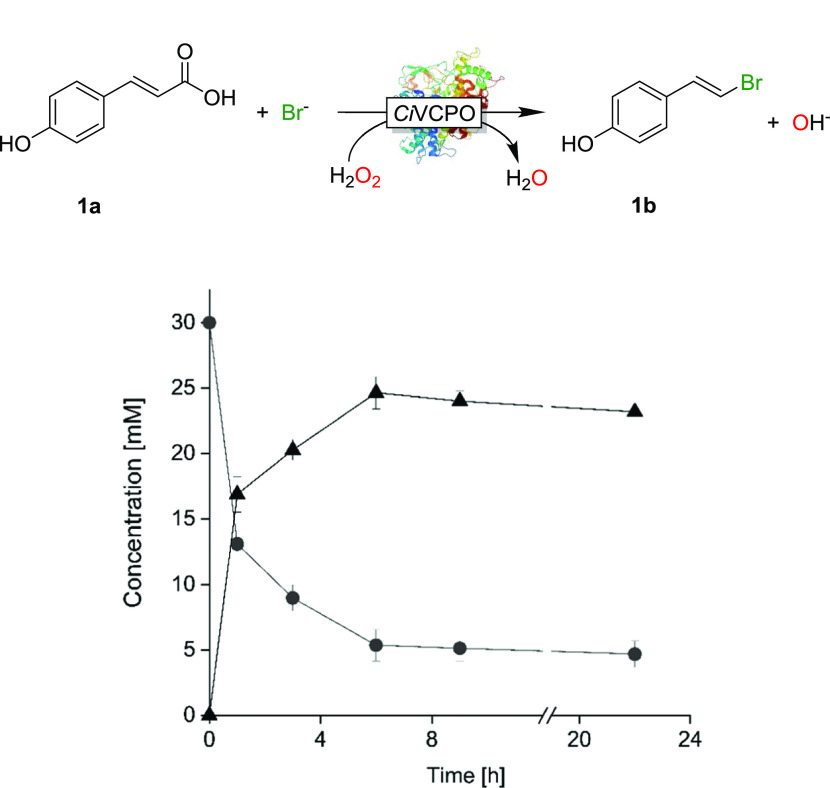
Time course of the chemoenzymatic decarboxylation of p-coumaric acid (●) (**1a**) to 4-(2-bromovinyl) phenol (▲) (**1b**). Conditions: [**1a**] = 30 mM, citrate buffer (100 mM, pH 5.0), [*Ci*VCPO] = 400 nM, [KBr] = 50 mM, [H_2_O_2_] = 30 mM, 5% dimethyl sulfoxide (DMSO), 30 °C, 1 mL. The data shown are the results from duplicate experiments.

The biocatalyst (*Ci*VCPO) was produced via heterologous expression in recombinant *Escherichia coli* following previously established procedures.^25^ Using *p*-coumaric acid (**1a**, 30 mM) as a model substrate, the desired product 4-(2-bromovinyl) phenol (**1b**) was readily obtained under the reaction conditions chosen initially ([*Ci*VCPO] = 400 nM, [KBr] = 50 mM, [H_2_O_2_] = 30 mM, Figure 1). An initial reaction rate of 6.97 mM h^–1^ was observed (corresponding to a catalytic turnover frequency of the biocatalyst of 4.8 s^–1^). After approx. 6 h, a final yield of 82% (gas chromatography, GC yield) was obtained corresponding to 61,600 turnover number (TON) for *Ci*VCPO. The reaction could be scaled up to 50 mL, resulting in 58% isolated yield (173 mg, Figures S1–S3). All relevant negative controls (i.e., performing the reaction in the absence of either *Ci*VCPO or H_2_O_2_ or using thermally inactivated *Ci*VCPO) failed to form any bromination products. Also substituting *Ci*VCPO with a 25-fold excess of NaVO_3_ (under otherwise identical reaction conditions) did not give any decarboxylated product (Table S1).

Next, we investigated some key parameters (enzyme concentration, pH, H_2_O_2_ and KBr concentration) influencing oxidative decarboxylation in more detail (Table 1). The reaction rate correlated with the enzyme concentration (Table 1, entries 1–3). Increasing the concentration of H_2_O_2_ had a slightly negative effect on the product formation (Table 1, entries 3, 7–9). On one hand, the H_2_O_2_ concentration applied was significantly higher than the reported K_M_(H_2_O_2_) value for *Ci*VCPO of ≪0.1 mM, which is why the catalytic activity of *Ci*VCPO can be considered as being independent of the H_2_O_2_ concentration applied in these experiments. On the other hand, the rate of the hypobromite-initiated dismutation of H_2_O_2_^27^ increases at increasing H_2_O_2_ concentrations and thereby decreases the *in situ* concentration of hypobromite and H_2_O_2_. In line with the reported pH optimum^25^ of *Ci*VCPO, the highest catalytic rates were observed between pH 5 and 6 (Table 1, entries 3–6). An increase in the KBr concentration could lead to an increase in the reaction rate and product concentration (Table 1, entries 8 and 10), which we attribute to an increase in the *in situ* hypobromite concentration and the resulting acceleration of the chemical reaction step.

**Table 1:** Optimization of the Reaction Conditions^a^

entry	*c*(*Ci*VCPO) (nM)	pH	*c*(H_2_O_2_) (mM)	concn (mM)	initial rate^b^ (mM h^–1^)	TON^c^	selectivity^d^ (%)
1	100	5	30	10.3 ± 1.1	3.80	10,2700	99
2	200	5	30	14.6 ± 1.6	5.68	73,200	99
3	400	5	30	24.6 ± 1.2	6.97	61,600	97
4	400	4	30	10.9 ± 1.9	2.95	27,100	96
5	400	6	30	19.6 ± 1.0	6.49	48,880	96
6	400	7	30	12.3 ± 0.1	4.03	30,600	94
7	400	5	50	23.5 ± 5.7	6.36	58,700	98
8	400	5	100	19.4 ± 0.4	3.14	48,000	98
9	400	5	200	21.6 ± 3.5	5.76	54,000	98
10	400	5	100^e^	26.0 ± 0.7	7.95	65,000	97

a Reaction conditions: [*p*-coumaric acid] = 30 mM, citrate buffer (100 mM, pH 4–5) or NaPi buffer (100 mM, pH 6−7), [*Ci*VCPO] = 100–400 nM, [KBr] = 50–100 mM, [H_2_O_2_] = 30–200 mM, 30 °C, 5% DMSO, 6 h, 1 mL.

b The initial rate is based on concentration of **1b** at 3 h.

c TON = Turnover number ([**1b**]/[*Ci*VCPO]).

d The selectivity was determined by gas chromatography–mass spectrometry (GC–MS). Selectivity = [**1b**]/([**1b**] + [**1c**]) × 100%.

e [KBr] = 100 mM. A duplicate experiment was performed.

The highest formal *Ci*VCPO activity observed in these experiments (i.e., initial rate divided by the biocatalyst concentration) was 10.5 s^–1^ (Table 1, entry 1), which is in line with *Ci*VCPO activities previously observed (under comparable reaction conditions) ranging from 8.7 s^–1^ (in the case of Achmatowicz-type reactions)^28^ and 75 s^–1^ (as observed in the oxidative decarboxylation of glutamic acid).^23^ Bearing the chemoenzymatic character of these reactions in mind, the apparent differences in the formal *Ci*VCPO activity most likely originate from different reactivities of the chemical starting materials with OBr^–^, suggesting the chemical step of the reaction sequence being overall rate-limiting.

**Scheme 2:**
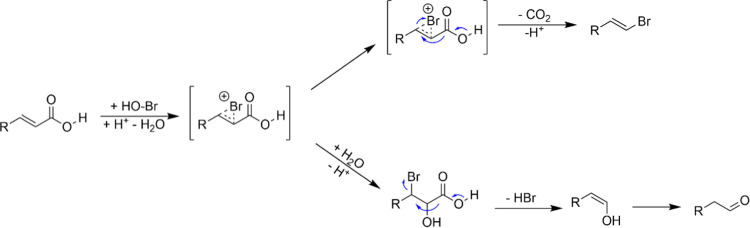
Proposed Nucleophilic Attack of Water to the Intermediate Bromonium Ion Competing with Its Decarboxylation

It should be noted that in all experiments, some formation of *p*-hydroxyphenylacetaldehyde (**1c**, Figure S4, ranging between 0.04 and 0.81 mM corresponding to 0.3–6.2%) was observed. Presumably, nucleophilic attack of water to the intermediate bromonium ion leading to the aldehyde product was observed (Scheme 2).

As a phenolic staring material, some ring halogenation was expected to occur.^29^ Interestingly, only upon prolonged reaction times, traces of the ring-brominated vinyl bromide product were observed in the case of decarboxylation of **1a** (Figure S4). Apparently, the conjugated C=C double bond reacted more readily than the aromatic ring system.

Next, we evaluated the substrate scope of the chemoenzymatic Hunsdiecker reaction in a 1.5 mmol scale by screening some commercially available substrates (Figure 2). Both substituted and nonsubstituted α,ß-unsaturated carboxylic acids could be transformed into the corresponding vinyl bromide products with good isolated yield (Figures S5–S37 and Table S2). Especially electron-donating substituted styrene derivates turned out to be good starting materials. Aromatic rings containing electron-withdrawing substituents such as halides, CN, CF_3_, or NO_2_ were not converted and the staring material was recovered. Also, for aliphatic α,ß-unsaturated carboxylic acids, no conversion was detectable under the experimental conditions applied here, which is in line with a previous report using *Cf*CPO.^20^

**Figure 2:**
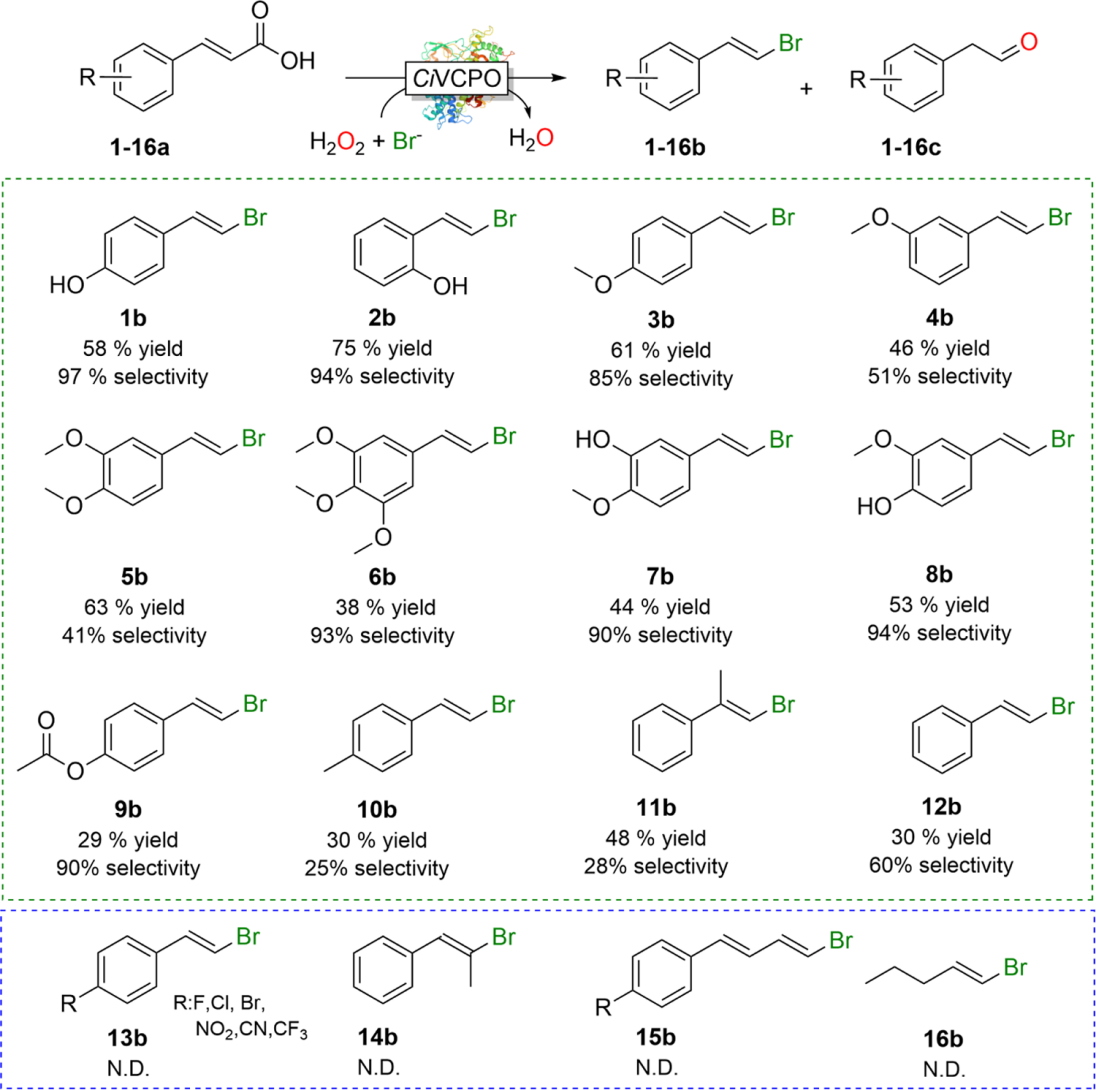
Substrate scope of preparative-scale chemoenzymatic decarboxylative bromination reaction. Conditions: [substrates] = 30 mM, citrate buffer (100 mM, pH 5), [*Ci*VCPO]= 400 nM, [KBr] = 50 mM, [H_2_O_2_] = 30 mM, 30 °C, 10 h, 50 mL scale. 5–20% DMSO to improve the substrate solubility. Isolated yield was calculated after the purification. The selectivity was determined by GC–MS using 5% DMSO in the reaction. Yield means isolated yield. Selectivity = ([**1**–**12b**])/([**1**–**12b**] + [**1**–**12c**]) × 100%. ND = not detected.

We found no obvious correlation between the substitution pattern of the aromatic substituent with the selectivity (halide vs aldehyde product).

**Figure 3:**
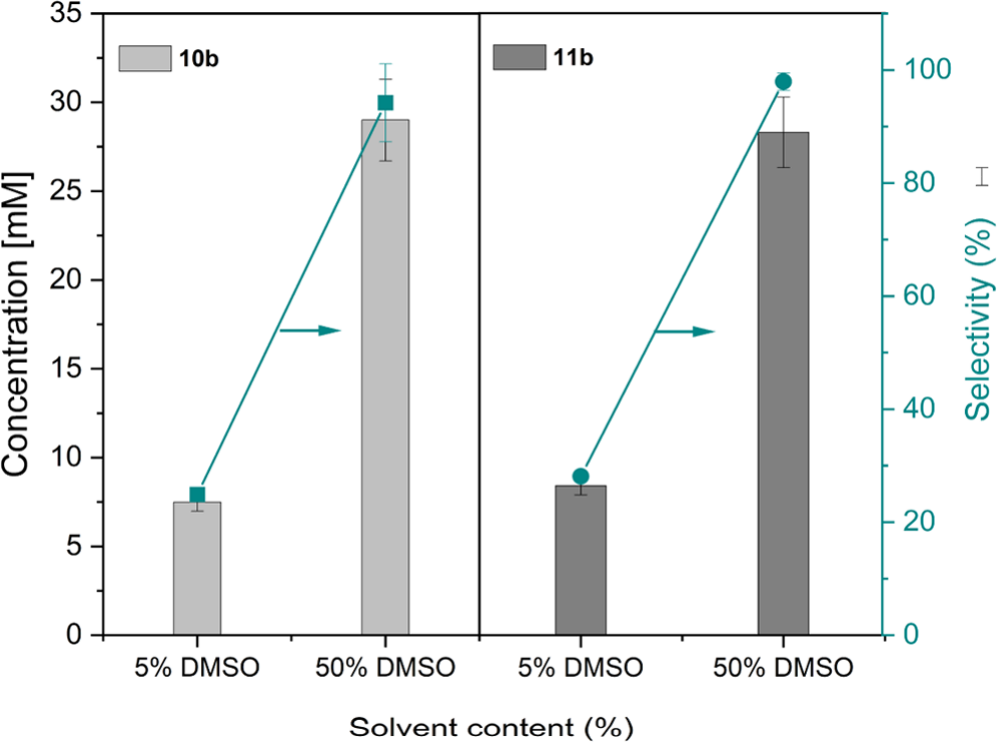
Dependence of the selectivity on the solvent content. Conditions: [substrates] = 30 mM, citrate buffer (100 mM, pH 5), [*Ci*VCPO]= 400 mM, [KBr] = 50 mM, [H_2_O_2_] = 30 mM, 30 °C, 6 h, 5 and 50% DMSO. A duplicate experiment was performed.

As shown in Figure 2, the vinyl bromide selectivity was rather poor in some cases. Based on the mechanistic proposal (Scheme 2), we hypothesized that the water activity may play a decisive influence on the vinyl bromide/aldehyde selectivity. To test this, we performed a range of experiments increasing the cosolvent concentration (DMSO) from 5% (v/v) to 50% (v/v) (Figure 3). Indeed, this approach proved successful increasing of the selectivity for **10b** and **11b** from roughly 25 to 95% (see also Figures S38 and S39 for **10a** and Figures S40 and S41 for **11b**). Also, other cosolvents such as methanol, isopropanol, or acetone had similar effects. We therefore concluded that medium engineering represents an excellent handle to control the selectivity of the oxidative decarboxylation.

**Figure 4:**
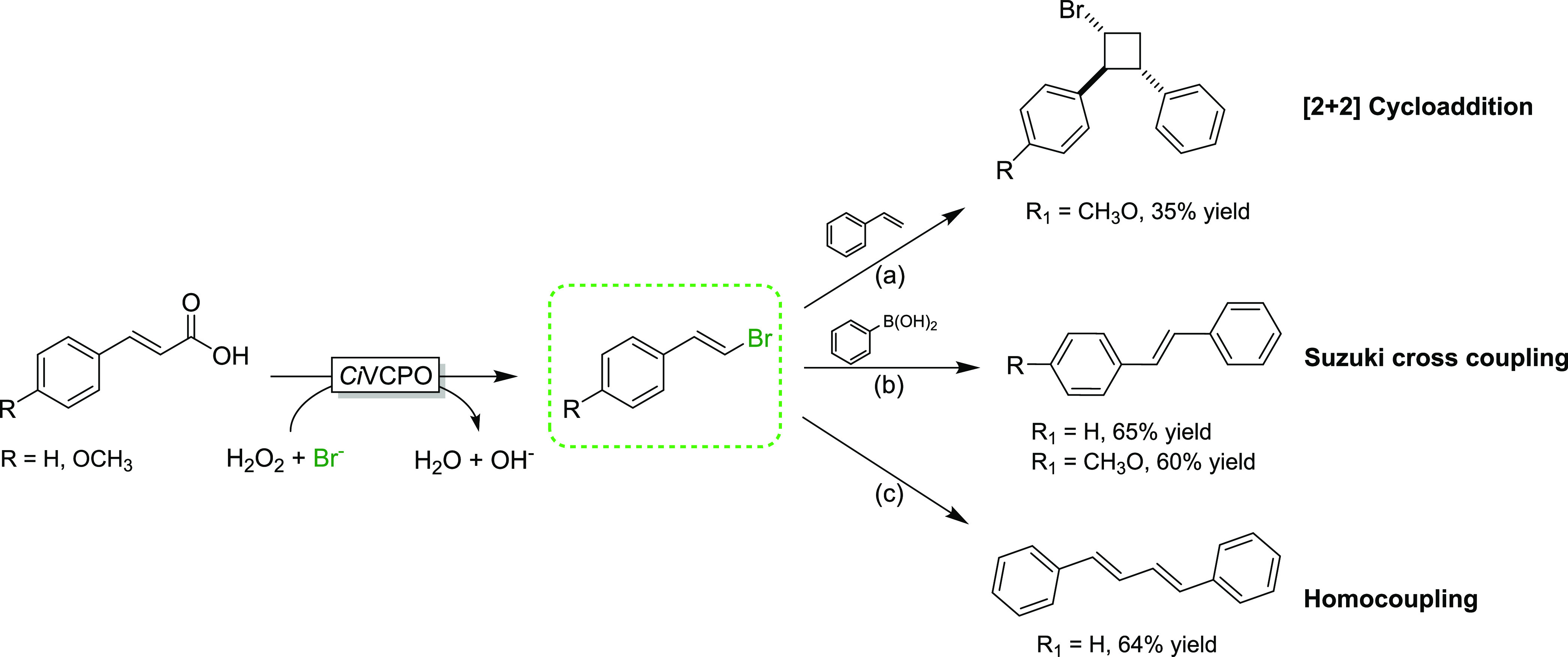
Expansion of chemoenzymatic Hunsdiecker-type reactions. Reaction conditions: (a) substrate = 0.22 mmol, styrene = 2.2 mmol, [TXT, (9-(2-methylphenyl)-1,3,6,8-tetramethoxythioxanthylium trifluoromethanesulfonate)] = 3 mol %, CH_3_CN, room-temperature (RT), air, green light-emitting diode (LED), 24 h; (b) [substrate] = 0.1–0.3 mmol, [phenyl boric acid] = 0.12–0.36 mol, [Pd(OAc)_2_] = 3 mol %, [orotic acid] = 6 mol %, [Cs_2_CO_3_] = 0.5 mmol, acetone, 100°C, N_2_, 16 h; and (c) [substrate] = 1 mmol, [Pd(OAc)_2_] = 0.02 mmol, Agarose = 0.05 g, [NaOH] = 1.5 mmol, H_2_O, 90°C, 12 h.

Finally, we explored the synthetic potential of the vinyl bromides obtained from the chemoenzymatic Hunsdieker reaction. For this, we submitted the products **3b** and **12b** to a photocatalytic [2 + 2] cycloaddition reaction with styrene,^30^ the Suzuki–Miyaura cross-coupling reaction with phenyl boronic acid,^31^ and a Pd-catalyzed Ullmann homocoupling reaction^32^ (Figure 4). In all cases, acceptable isolated yields of the desired products were obtained (for details, see the Supporting Information, Figures S43–S52).

## Conclusions

Overall, we have shown that vanadium chloroperoxidase from *C. inaequalis* is a robust catalyst for the oxidative decarboxylation of a broad scope of α,β-unsaturated carboxylic acids, establishing a chemoenzymatic Hunsdiecker reaction.

The selectivity of the reaction can be controlled by medium engineering, giving access to either the aldehyde or the vinyl bromide product.

The high activity and selectivity of the reaction and the mild and clean reaction conditions make the reaction attractive for the synthesis of valuable α,β-unsaturated halides from readily available starting materials.

## Supporting Information Available

Experimental details, enzyme preparation, ^1^H and ^13^C NMR, GC–MS, and control experiments (PDF)
