# Supplementary material for: Chemoenzymatic Hunsdiecker-Type Decarboxylative Bromination of Cinnamic Acids
Source: ACS Catal. 2022 Apr 4;12(8):4554–9. doi: 10.1021/acscatal.2c00485 (PMC9016706; doi:10.1021/acscatal.2c00485)
Supplement: Supplementary file 1 — cs2c00485_si_001.pdf [file cs2c00485_si_001.pdf]

Supporting Information

A Chemoenzymatic Hunsdiecker-type Decarboxylative Bromination of Cinnamic acids

Huanhuan Li,[1,4] Sabry H. H. Younes,[2,3] Shaohang Chen,[4] Peigao Duan,[1]* Chengsen Cui,[4,5] Ron Wever,[6] Wuyuan Zhang, [4,5]* Frank Hollmann[2]*

[1] School of Chemical Engineering and Technology, Xi'an Jiaotong University, Xi'an 710049, China;
[2] Department of Biotechnology, Delft University of Technology, Van der Maasweg 9, 2629HZ Delft, The Netherlands.
[3] Department of Chemistry, Faculty of Sciences, Sohag University, Sohag 82524, Egypt.
[4] Tianjin Institute of Industrial Biotechnology, Chinese Academy of Sciences, 32 West 7th Avenue, Tianjin 300308, China
[5] National Center of Technology Innovation for Synthetic Biology, 32 West 7th Avenue, Tianjin 300308, China
[6] Van't Hoff Institute for Molecular Sciences, University of Amsterdam, 1098 XH Amsterdam, The Netherlands

* E-mail: pgduan@xjtu.edu.cn; zhangwy@tib.cas.cn; f.hollmann@tudelft.nl

Number of pages: 63

Number of tables: 2 (Tables S1 and S2)

Number of figures: 52 (Figure S1-S52)

Table of Contents

Table of Contents 2
General information 3
Preparation of Vanadium-Chloroperoxidase 3
Purification of the Enzymes 3
The general procedures of the decarboxylation to synthesize vinyl halides 4
Semi-preparative synthesis of halogenated ether compounds 4
NMR and GC-MS spectrum (1b-12b) 6
The synthesis of TXT catalyst 46
The general procedure for crossed intermolecular cyclic [2+2] cycloaddition 49
The general procedure Suzuki–Miyaura cross-coupling reaction 52
The general procedure of Homocoupling reaction 57
References 63

General information

Materials

All chemicals and reagents were obtained from commercial suppliers (Sigma-Aldrich, Bide Pharmatech Ltd., Alfa Aesar, Macklin, Energy chemical, etc.) and used without further pre-treatments.

Preparation of Vanadium-Chloroperoxidase

The heterologous expression and purification of vanadium-chloroperoxidase from *Curvularia inaequalis* (*Ci*VCPO) were performed according to our reported procedures[1]. A 2 L culture of *Escherichia coli* transformant (*E. coli* TOP10 (Invitrogen) with the construct *pBAD-VCPO*) was grown at 37 °C in LB medium supplemented with 100 µg/mL ampicillin to an OD 600 nm between 0.6 and 0.8. Protein expression was induced by adding 0.02 % L arabinose and kept the fermentation broth at 20 °C for 2 hours. The incubation then continued for 24 hours at 25 °C before cell harvest.

Purification of the Enzymes

Cells were harvested by centrifugation at 8000 rpm for 10 min at 4 °C (4.3xg). The cells were resuspended to 1 g mL^{-1} in 50 mM Tris/H_2SO_4, pH 8.1 fortified with protease inhibitors, lysozyme (2 mg mL^{-1}) and DNaseI. Cells were lysed using a cell disruptor and debris was removed by centrifugation at 15000 rpm for 1 h at 4 °C. Then an equal volume of isopropyl alcohol was added to the supernatant to precipitate nucleic acids and unstable proteins. After centrifugation (30 min at 15000 rpm), the clear supernatant was applied to a DEAE Sephacel column (Amersham Pharmacia Biotech) (5mL min^{-1}) equilibrated with 50 mM Tris/H_2SO_4 (pH 8.1). After washing the column with 2 volumes of 50 mM Tris/ H_2SO_4 (pH 8.1), and 2 volumes of 0.1 M NaCl in 50 mM Tris/H_2SO_4 (pH 8.1), the enzyme was eluted with 1 M NaCl in 50 mM Tris/HCl. Finally, the pure apoenzyme was dialysed against 100 µM orthovanadate in 50 mM Tris H_2SO_4, pH 8.1 to obtain the reconstituted holoenzyme. The protein concentration was estimated by the BSA assay.

General procedure for the decarboxylation to synthesise vinyl halides

The α,β-unsaturated carboxylic acid (**1a-12a**, 30mM), H_2O_2 (30mM), KBr (50mM), and 400nM *Ci*VCPO were added in a 1mL citrate buffer solution (100mM, pH 5.0). The above concentration represents the final concentration of each ingredient. Then, the mixture was mixed and reacted under the 30°C in a thermal shaker with 800 rpm. The product concentration was obtained by using calibration curves in GC. To determine the selectivity, the integrated peaks of the vinyl product and aldehyde in GC-MS were used.

Semi-preparative synthesis of halogenated ether compounds

In a semi-preparative synthesis (50 mL), the same reaction conditions were applied as described above, except that the amount of the auxiliary solvent (DMSO) was increased to improve the substrate solubility. After reactions, the mixture was extracted with ethyl acetate three times and dried over Na_2SO_4. The solvent was evaporated at 45 °C and the products were purified by flash chromatography (Biotage rapid preparation system) using 5% - 20% ethyl acetate in petroleum ether. The isolated yield is calculated based on the amount of the vinyl halide products in comparison to the substrate added.

Analytics

GC analysis

Sample preparation: After the reaction, the reaction mixture was extracted using ethyl acetate containing dodecane as internal standard (5 mM) (extraction ratio: 1:2) and dried over Na_2SO_4, then, the sample were analysed by gas chromatography (GC).
Sample analysis: The GC data were analysed by SHIMADZU GC-2010 Pro equipped with column SH-Rtx-1 (30m × 0.25mm × 0.25μm). The temperature profile was 120 °C holding for 0.8min; 30 °C min^{-1} to 180°C holding for 1.2 min; 30 °C min^{-1} to 230°C for 1 min; 30 °C min^{-1} to 320°C for 0.5 min.

GC-MS analysis

Sample preparation: After the reaction, the reaction mixture was extracted using pure ethyl acetate and dried over Na_2SO_4. The sample were then analysed by gas chromatography-mass spectroscopy (GC-MS).
Sample analysis: Electron ionisation (EI) GC-MS data were collected on an Agilent model 7890A GC with a DB-5 fused silica capillary column (30 m length, 0.25 mm inner diameter, 0.25 μm film thickness), Agilent 7200 Q-TOF mass selective detector and

7683B autosampler. The GC was programmed from 60 °C (held for 1 min) to 150 °C at 20°C min^{-1} (held for 1 min); 20 °C min^{-1} to 220 °C holding for 1.5 min; 20 °C min^{-1} to 300 °C holding for 1.5 min; the injection port temperature was 250 °C, and the transfer line temperature was 280 °C using the following parameters: ultra-high purity helium carrier gas, column flow at 1 mL min^{-1}, injection port temperature 250 °C, transfer line temperature 280 °C. The MS were scanned through full-scan data acquisition from 35 to 550 atomic mass units.

NMR spectroscopy analysis

^{1}H NMR spectra were recorded at 298.2 K on a Bruker AVANCE III 400 MHz NMR spectrometer (Bruker Bio spin, Germany), operating at 400 MHz for proton frequency, and 101 MHz for carbon frequency. TMS was used an internal standard and CD_3Cl was used as the solvent.

NMR and GC-MS spectrum (1b-12b)

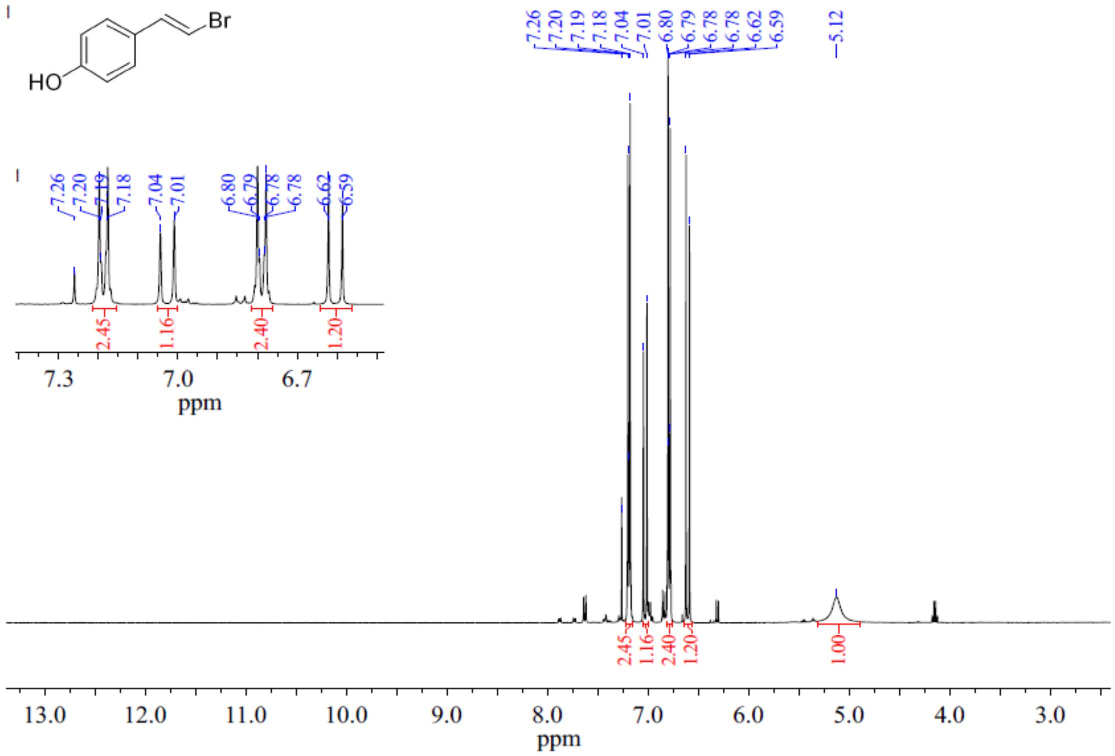

Figure S1. ^{1}H NMR spectrum of 4-(2-bromovinyl) phenol (**1b**) in $CDCl_3$.

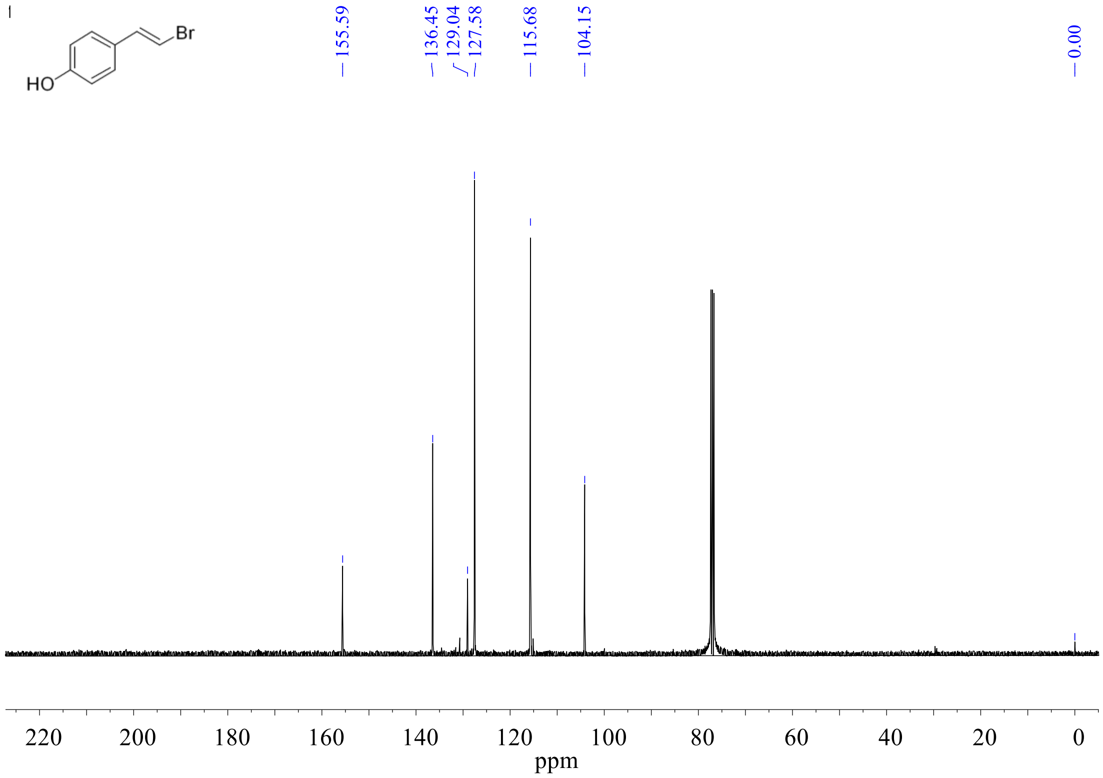

Figure S2. ^{13}C NMR spectrum of 4-(2-bromovinyl) phenol (**1b**) in $CDCl_3$.

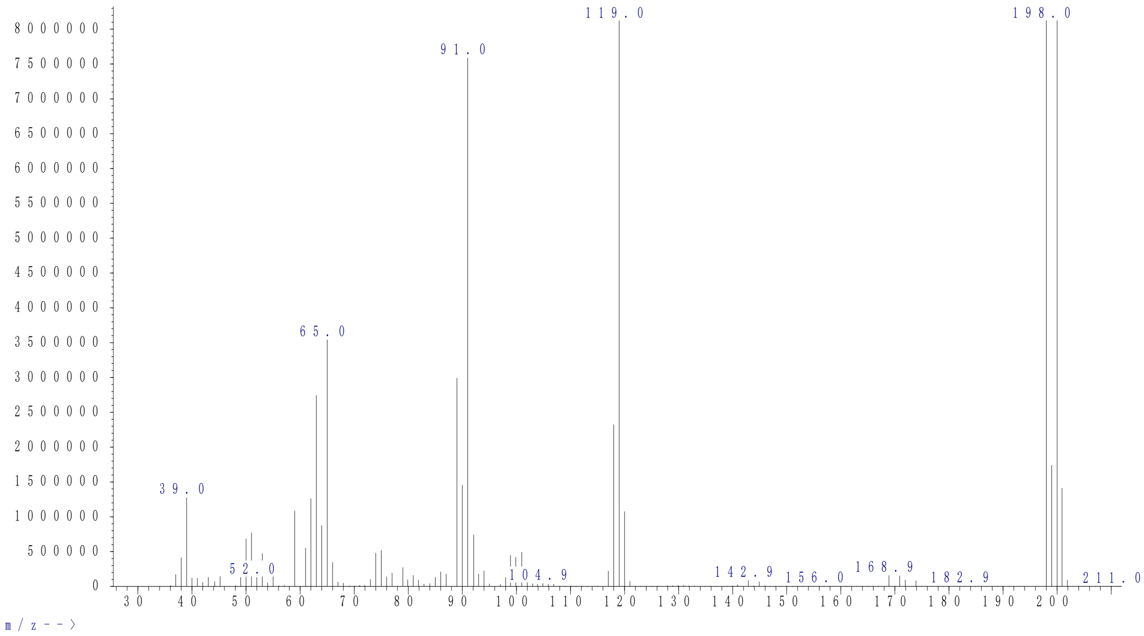

Figure S3. Mass spectrum of 4-(2-bromovinyl) phenol (**1b**) from GC-MS.

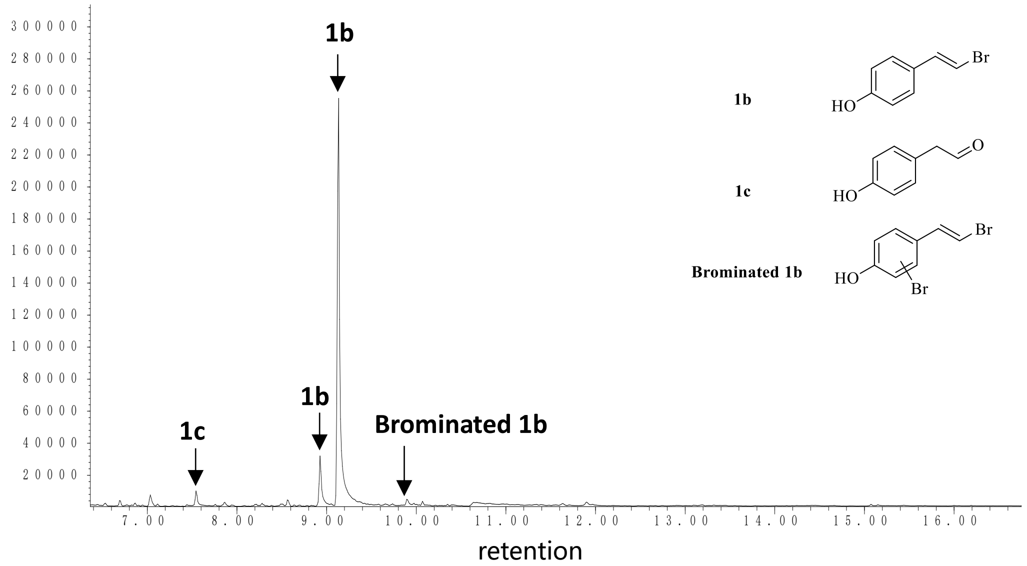

Figure S4. Representative GC-MS chromatogram of the reaction mixture of the decarboxylation of *p*-coumaric acid (**1a**) with 5% DMSO in 1mL scale.

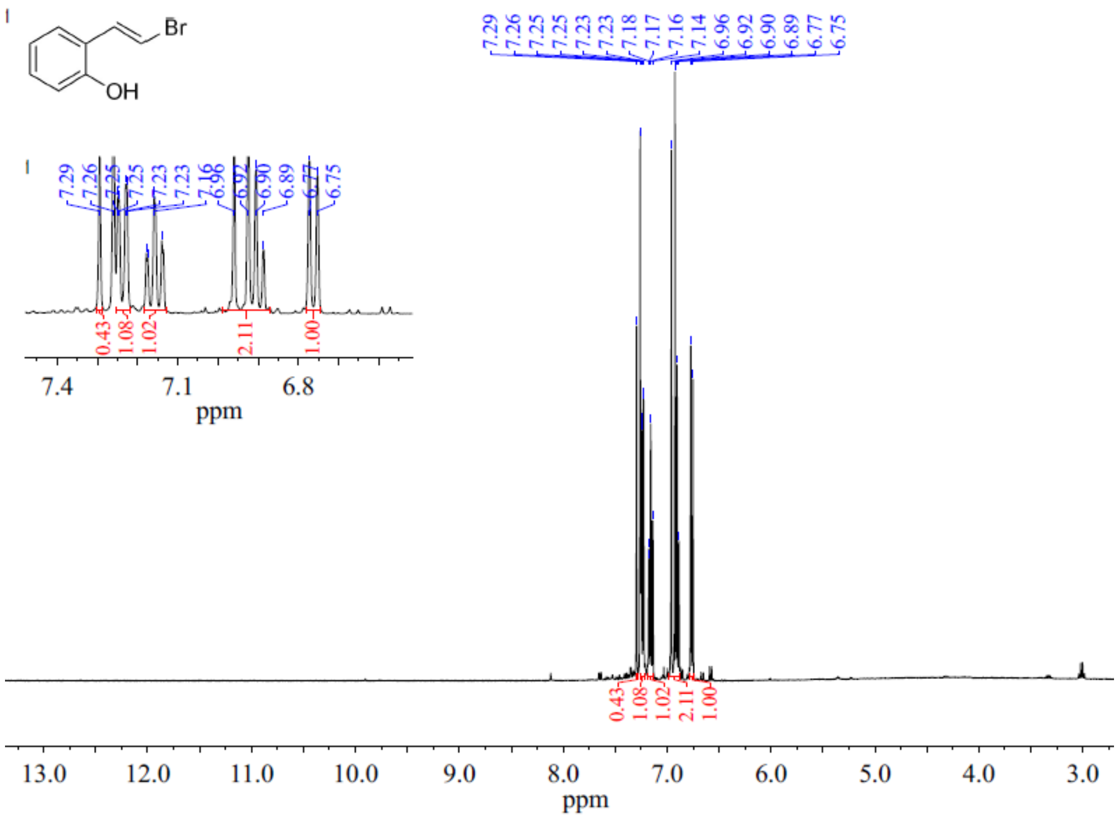

Figure S5. ^{1}H NMR spectrum of 2-(2-bromovinyl) phenol (**2b**) in $CDCl_3$.

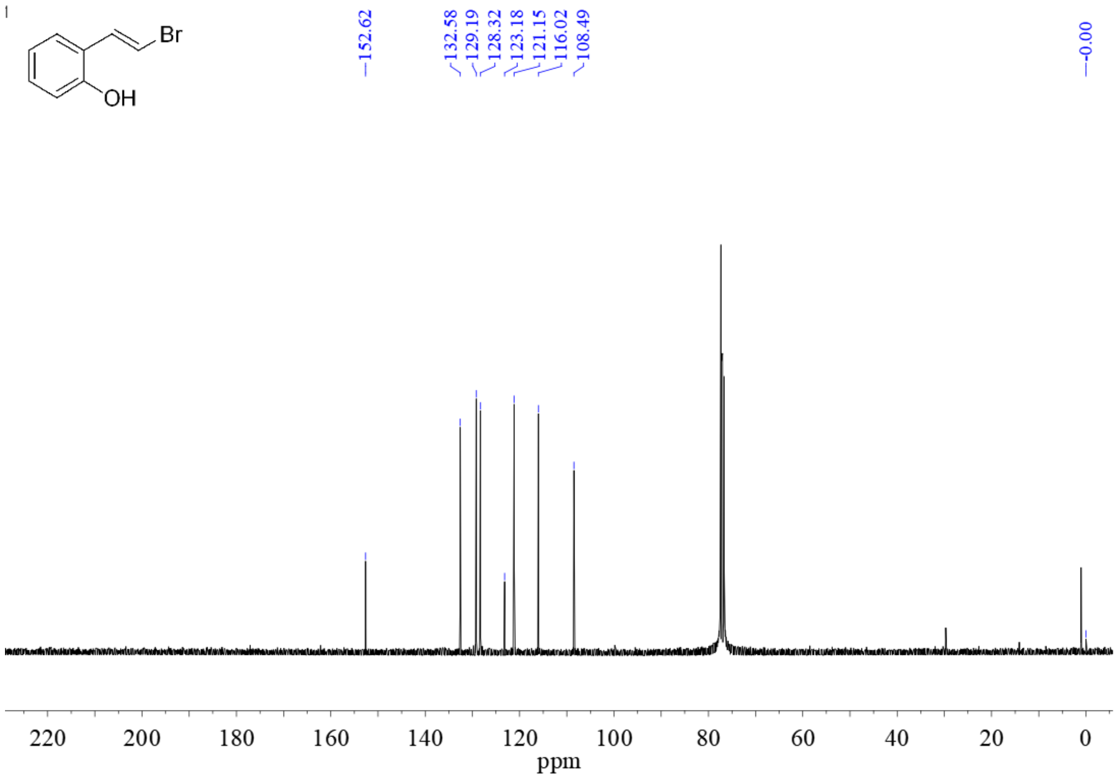

Figure S6. ^{13}C NMR spectrum of 2-(2-bromovinyl) phenol (**2b**) in $CDCl_3$.

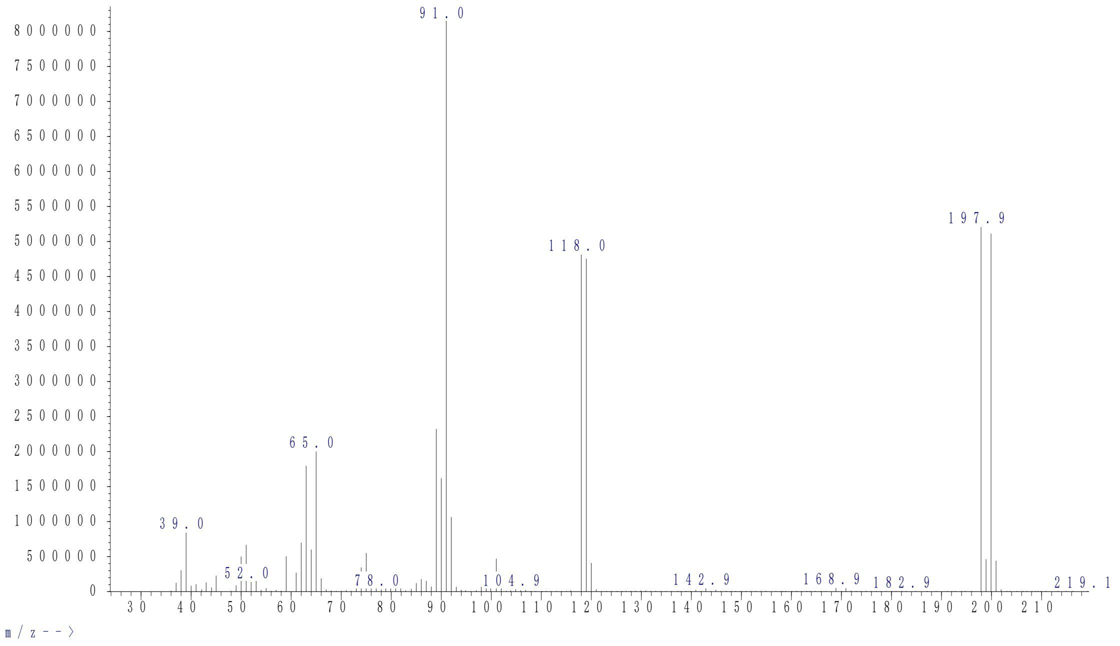

Figure S7. Mass spectrum of 2-(2-bromovinyl) phenol (**2b**) from GC-MS.

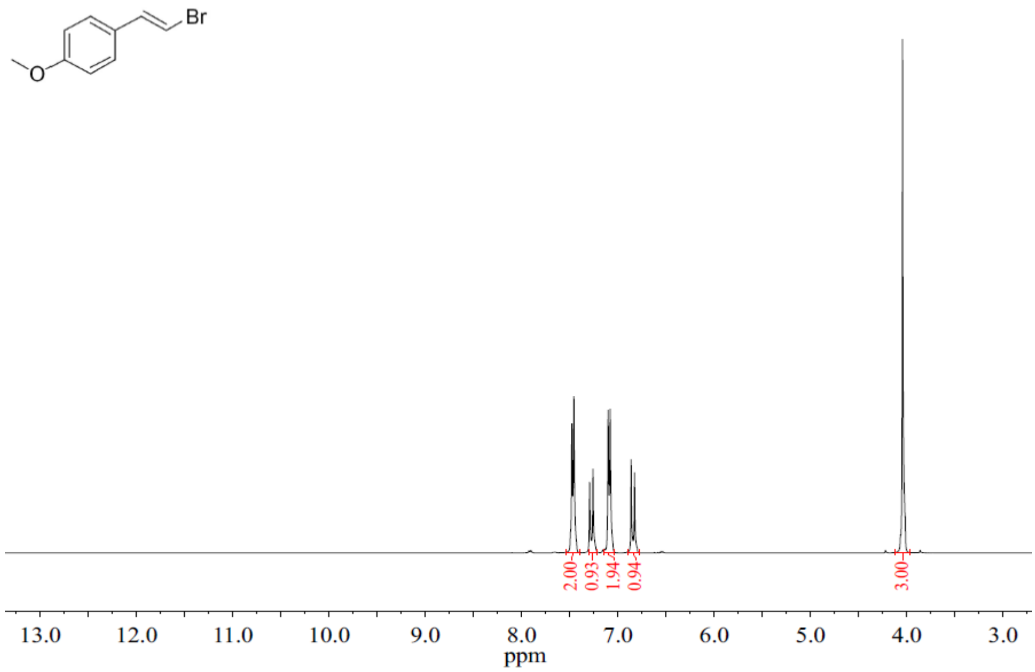

Figure S8. ^{1}H NMR spectrum of 1-(2-bromovinyl)-4-methoxybenzene (**3b**) in $CDCl_3$.

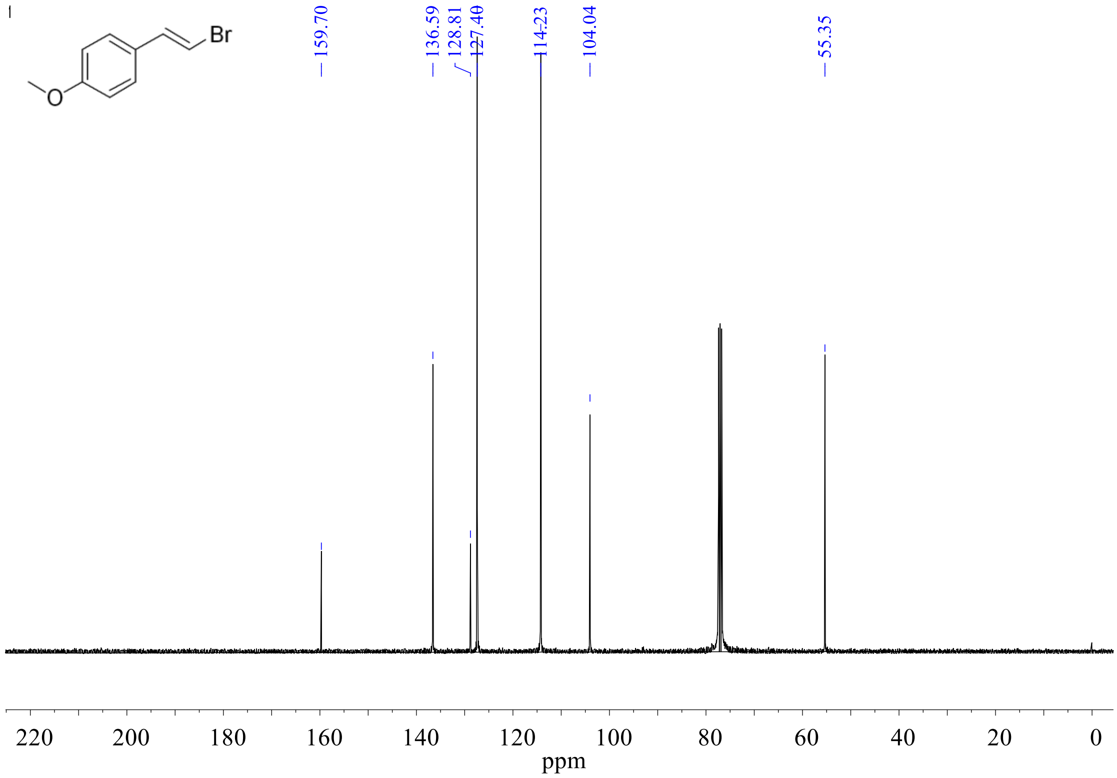

Figure S9. ^{13}C NMR spectrum of 1-(2-bromovinyl)-4-methoxybenzene (**3b**) in $CDCl_3$.

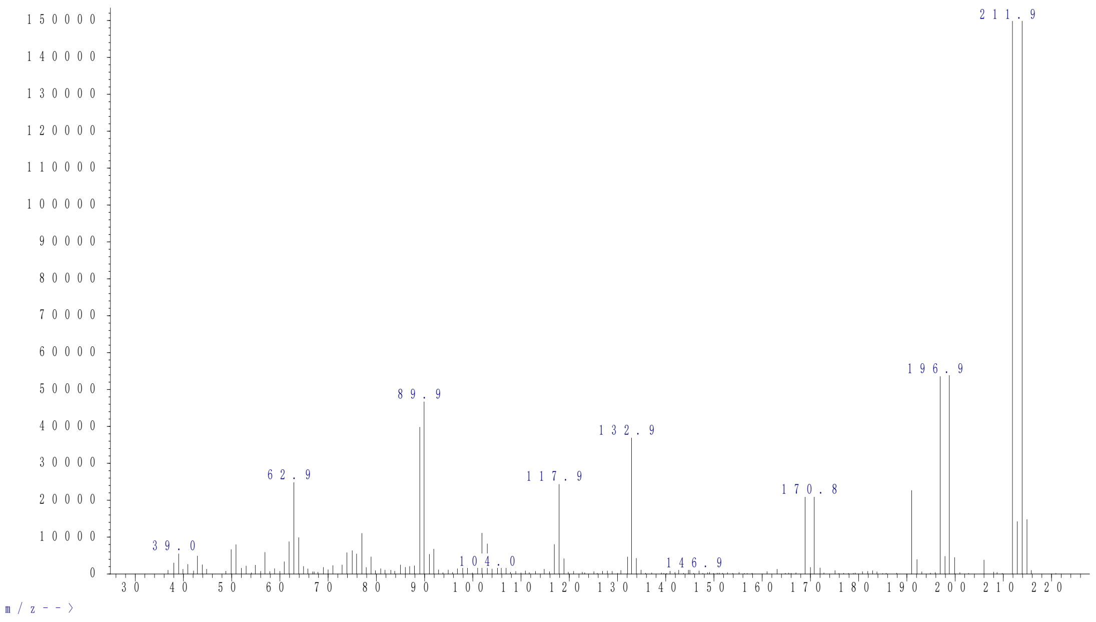

Figure S10. Mass spectrum of 1-(2-bromovinyl)-4-methoxybenzene (**3b**) in GC-MS.

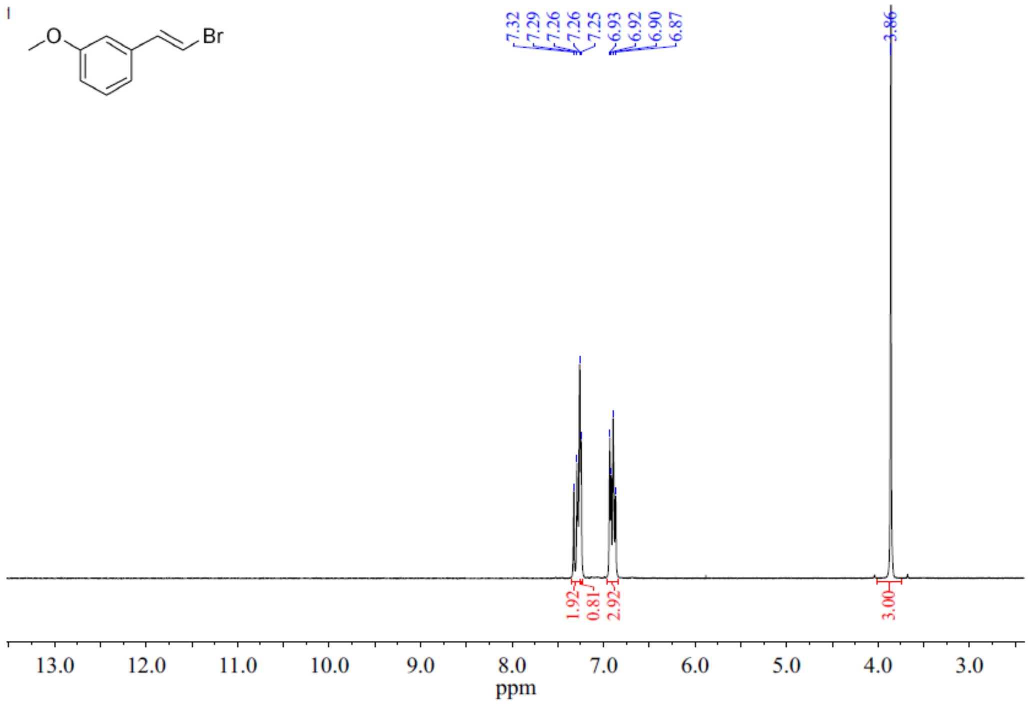

Figure S11. ^{1}H NMR spectrum of 1-(2-bromovinyl)-3-methoxybenzene (**4b**) in $CDCl_3$.

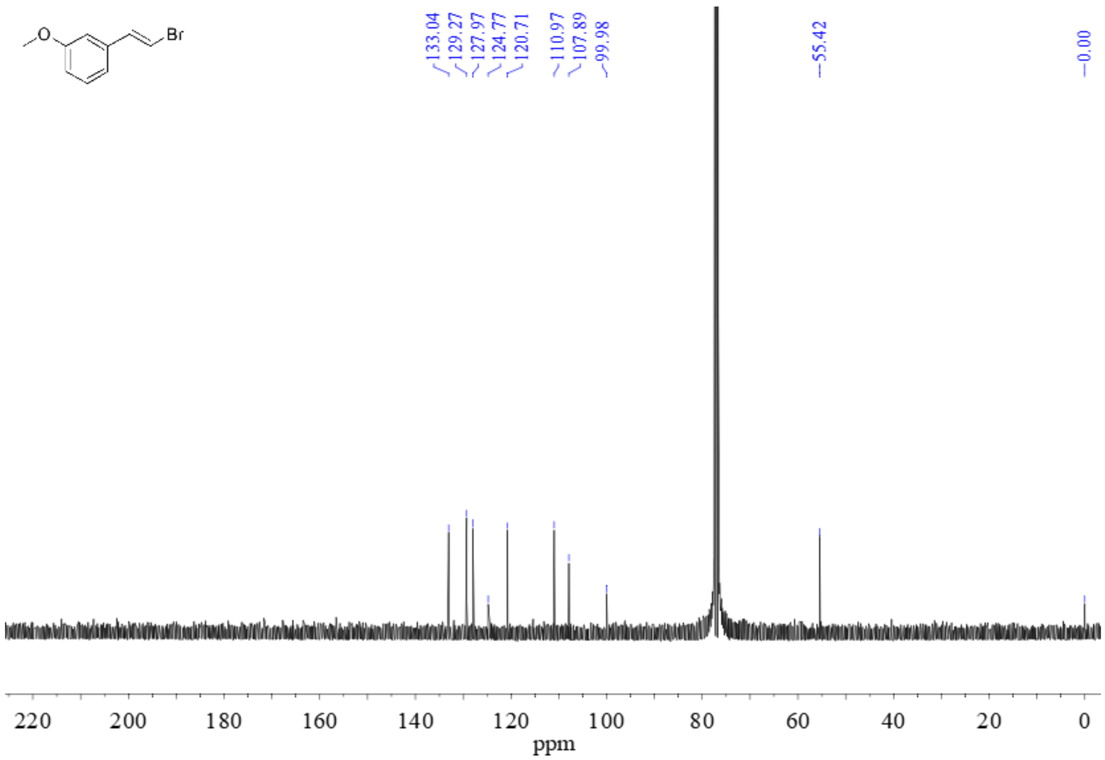

Figure S12. ^{13}C NMR spectrum of 1-(2-bromovinyl)-3-methoxybenzene (**4b**) in $CDCl_3$.

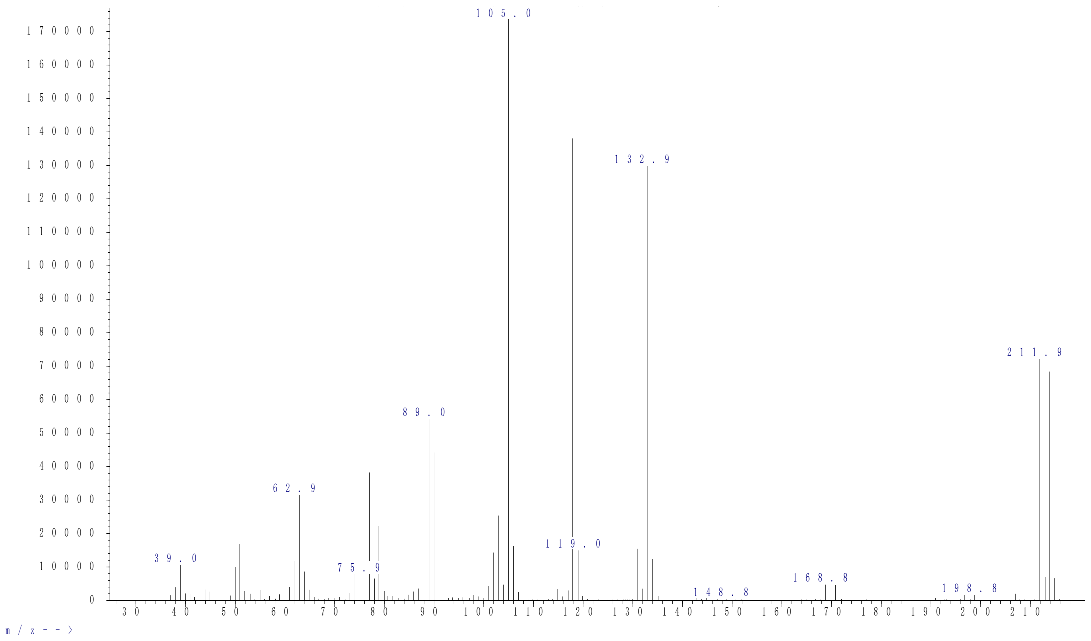

Figure S13. Mass spectrum of 1-(2-bromovinyl)-4-methoxybenzene (**4b**) in GC-MS.

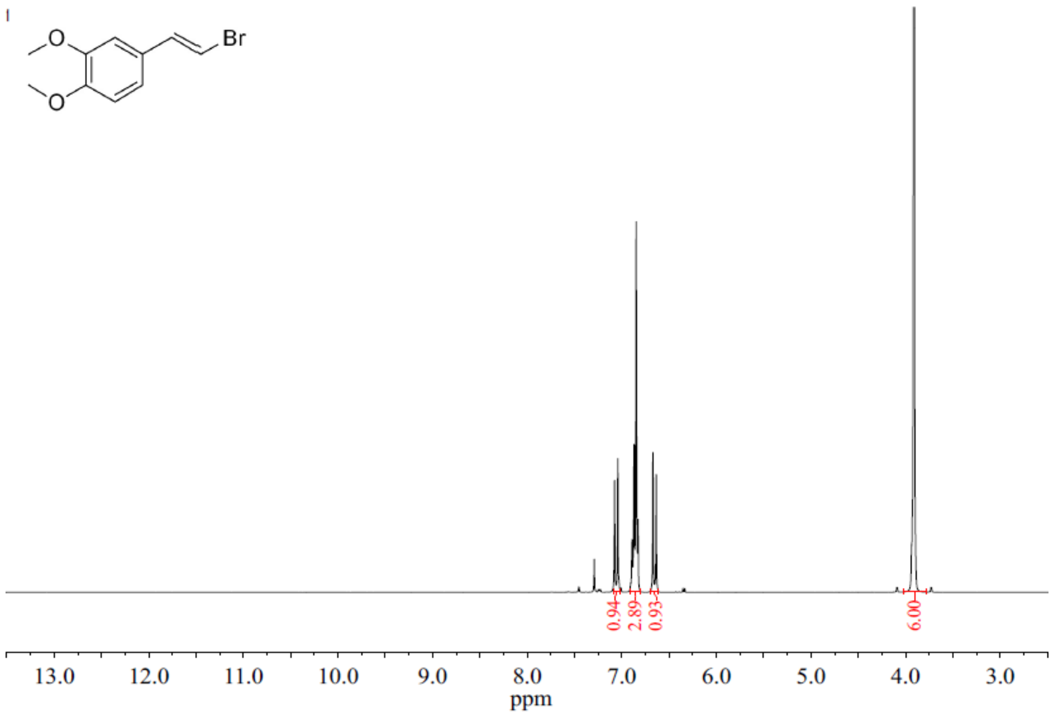

Figure S14. ^{1}H NMR spectrum of 4-(2-bromovinyl)-1,2-dimethoxybenzene (**5b**) in $CDCl_3$.

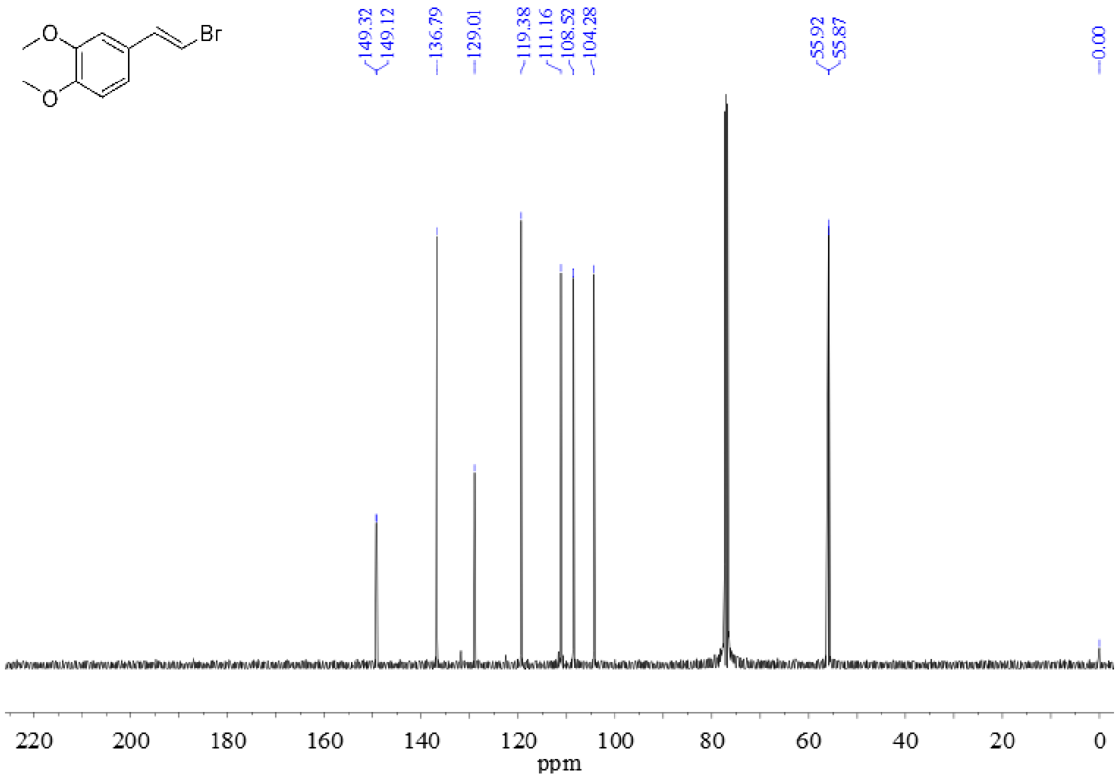

Figure S15. ^{13}C NMR spectrum of 4-(2-bromovinyl)-1,2-dimethoxybenzene (**5b**) in $CDCl_3$.

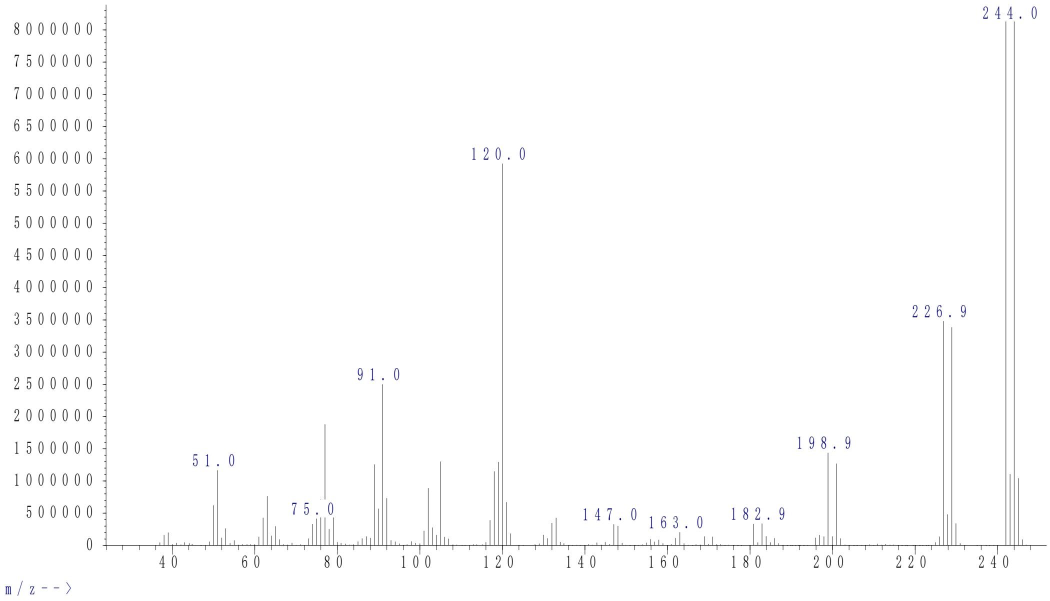

Figure S16. Mass spectrum of 4-(2-bromovinyl)-1,2-dimethoxybenzene (**5b**) in GC-MS.

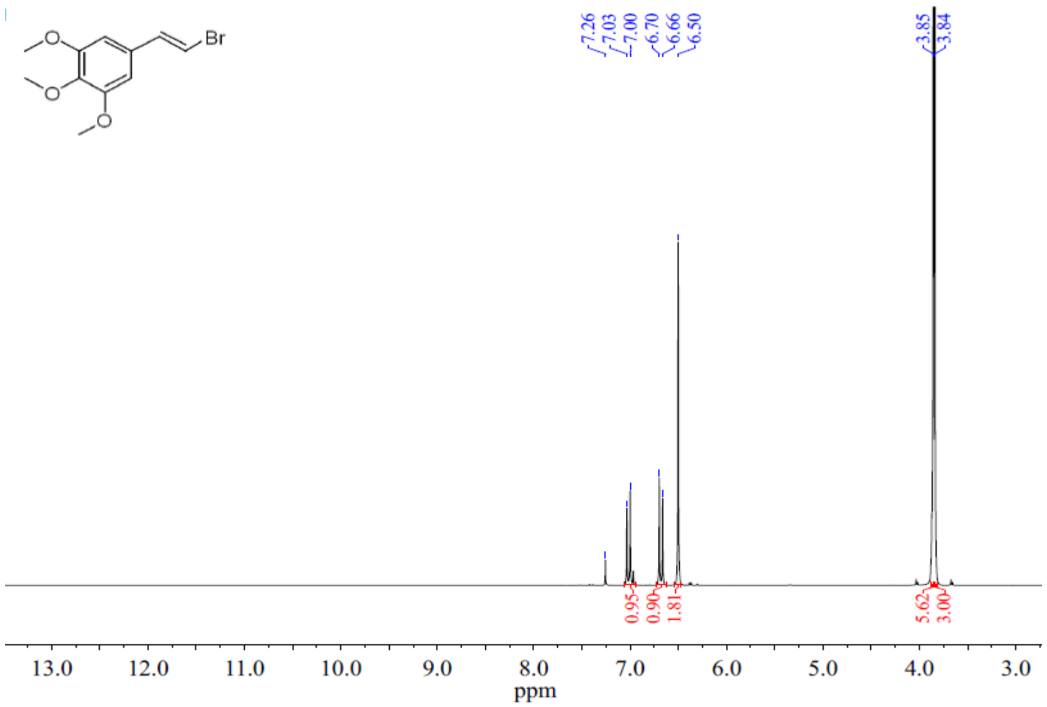

Figure S17. ^{1}H NMR spectrum of 5-(2-bromovinyl)-1,2,3-trimethoxybenzene (**6b**) in $CDCl_3$.

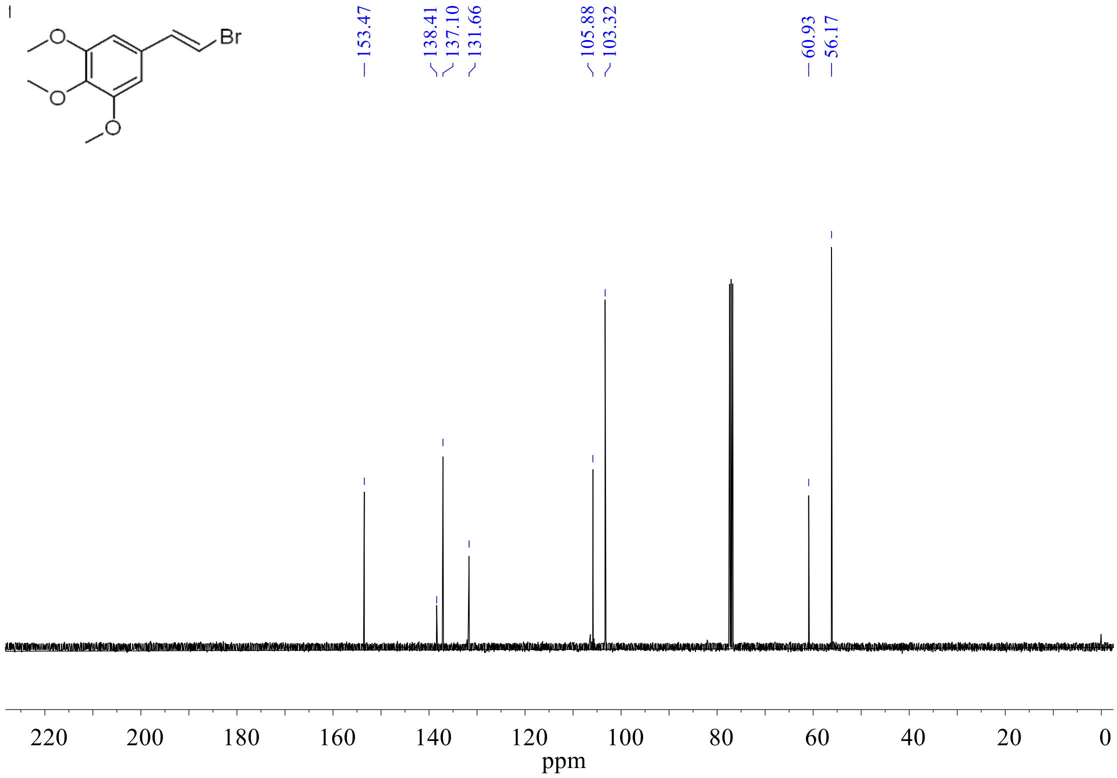

Figure S18. ^{13}C NMR spectrum of 5-(2-bromovinyl)-1,2,3-trimethoxybenzene (**6b**) in $CDCl_3$.

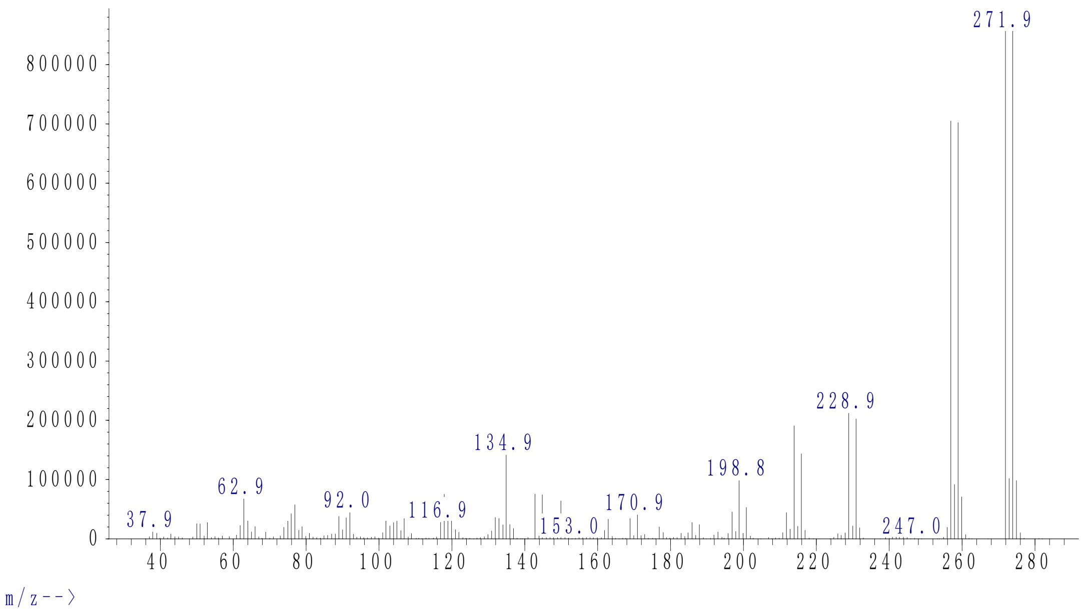

Figure S19. Mass spectrum of 5-(2-bromovinyl)-1,2,3-trimethoxybenzene (**6b**) in GC-MS.

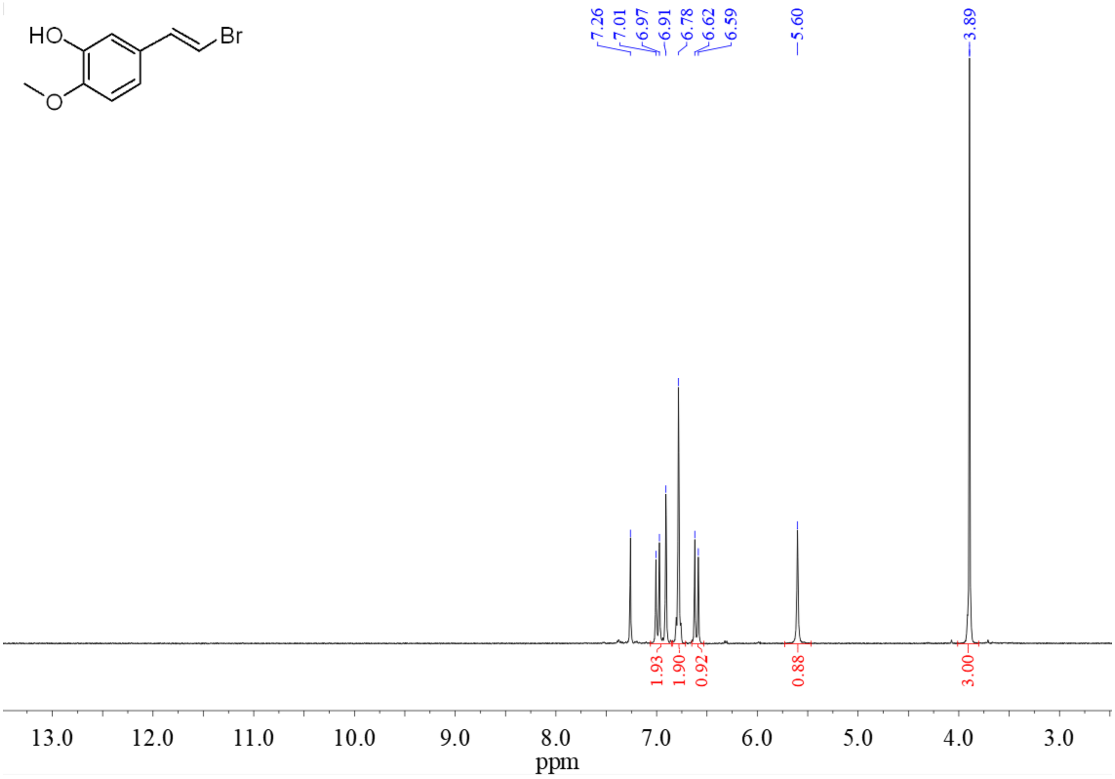

Figure S20. ^{1}H NMR spectrum of 5-(2-bromovinyl)-2-methoxyphenol (**7b**) in $CDCl_3$.

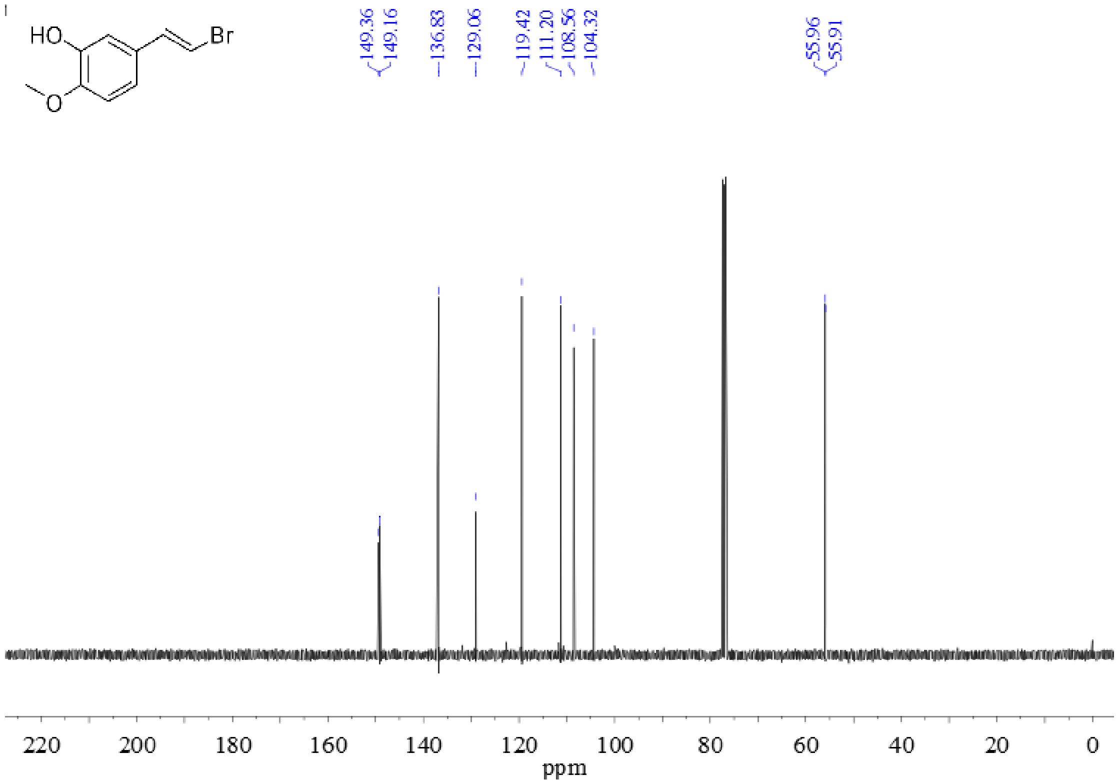

Figure S21. ^{13}C NMR spectrum of 5-(2-bromovinyl)-2-methoxyphenol (**7b**) in $CDCl_3$.

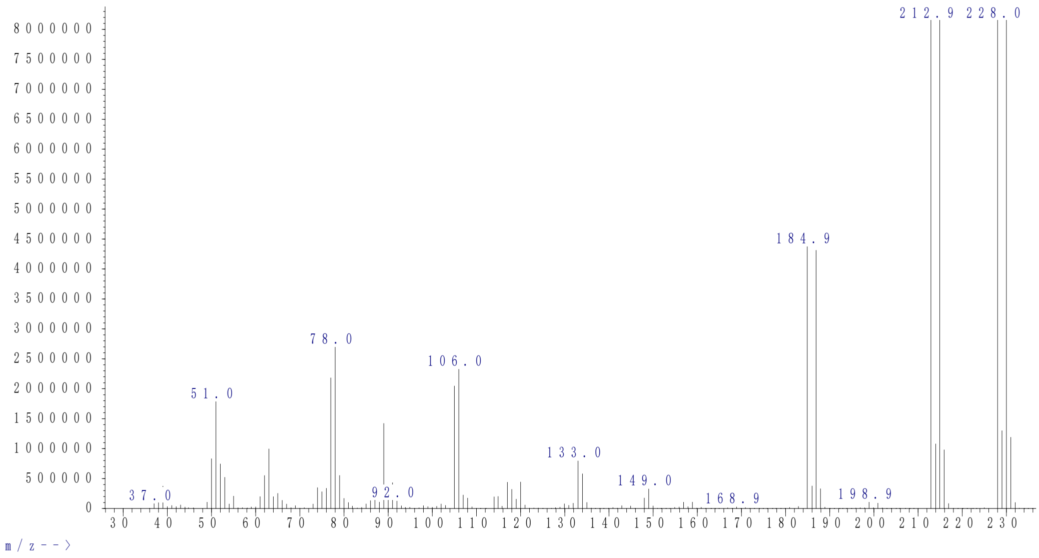

Figure S22. Mass spectrum of 5-(2-bromovinyl)-2-methoxyphenol (**7b**) in GC-MS.

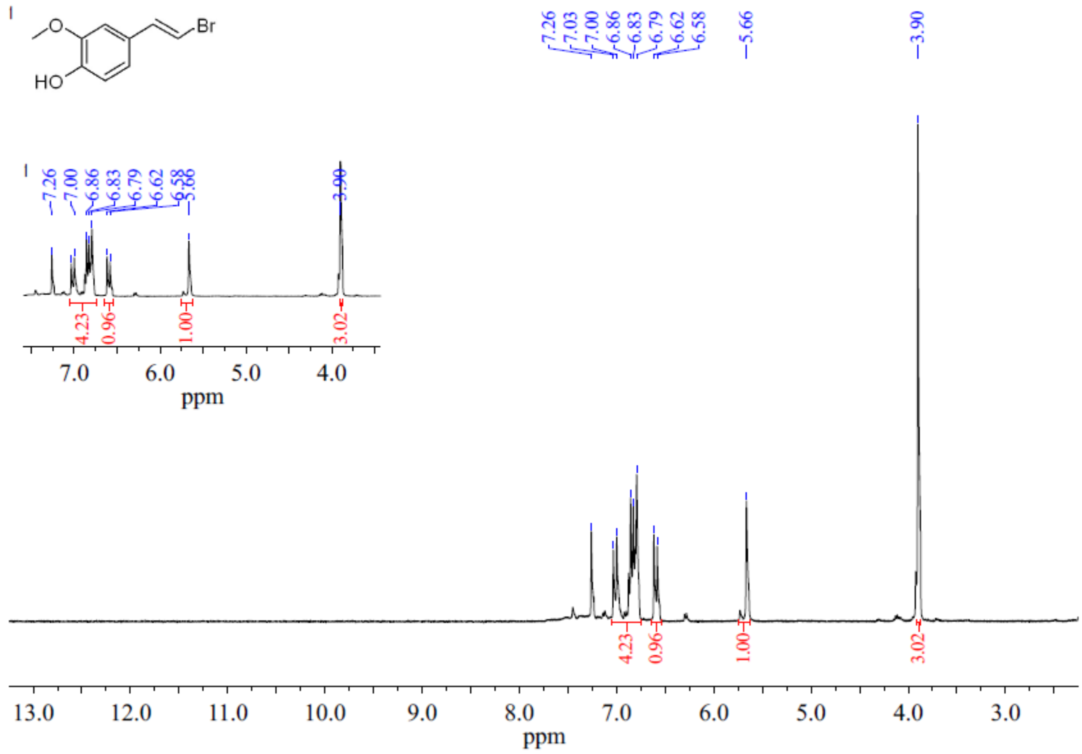

Figure S23. ^{1}H NMR spectrum of 4-(2-bromovinyl)-2-methoxyphenol (**8b**) in $CDCl_3$.

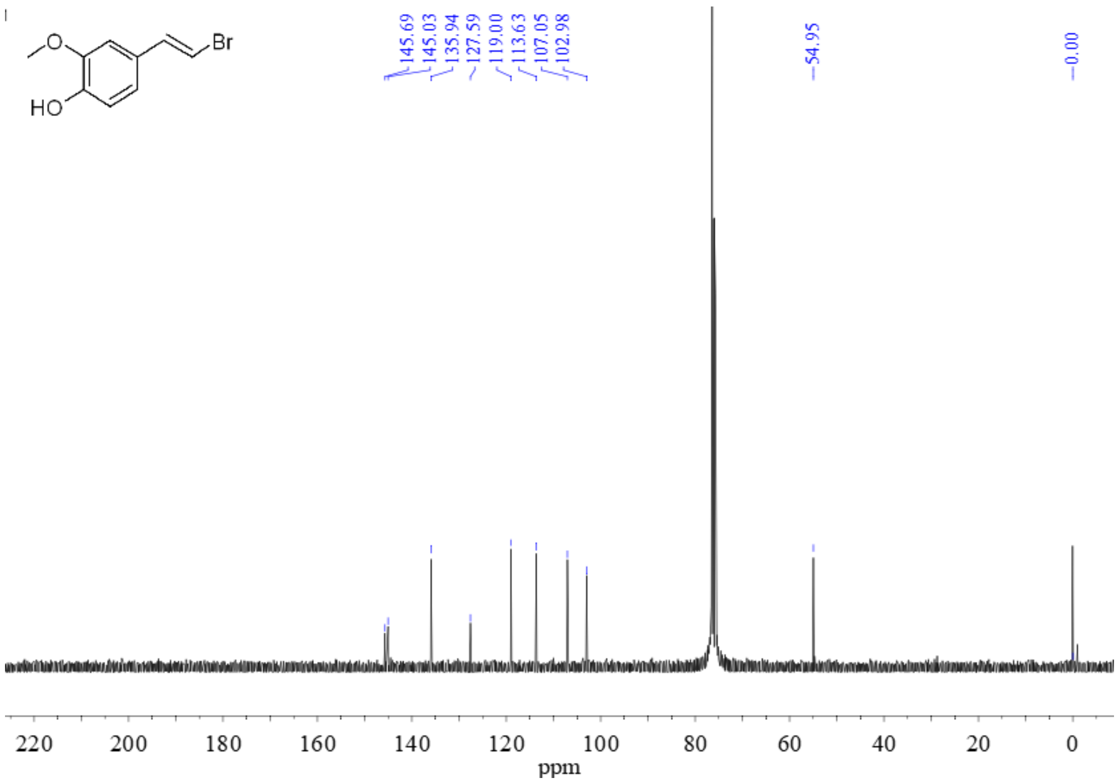

Figure S24. ^{13}C NMR spectrum of 4-(2-bromovinyl)-2-methoxyphenol (**8b**) in $CDCl_3$.

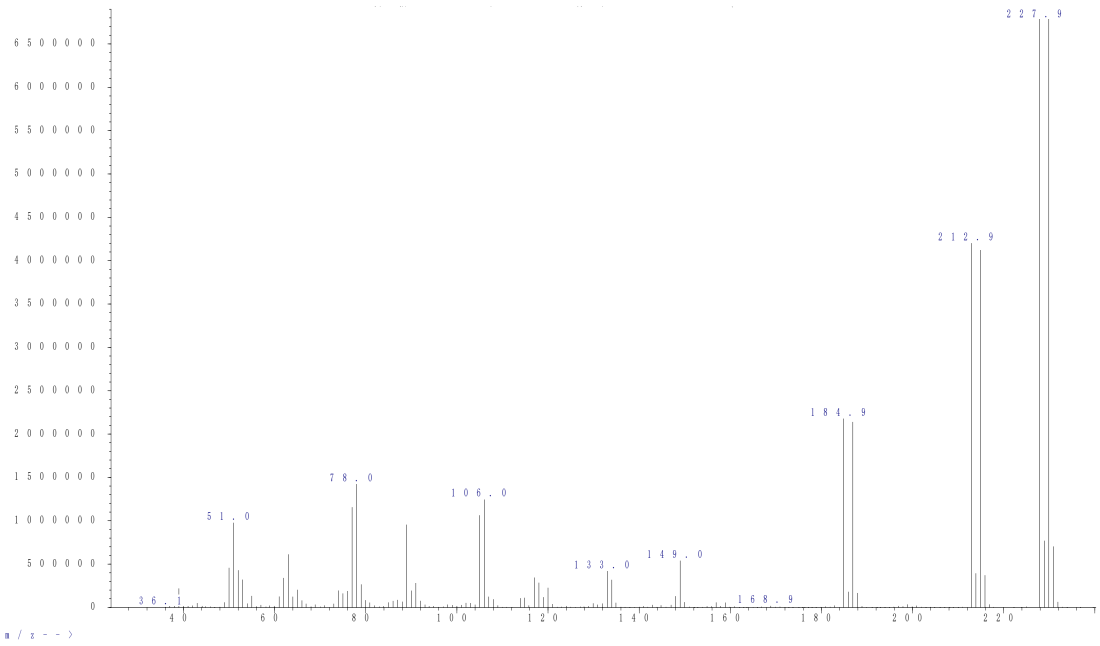

Figure S25. Mass spectrum of 4-(2-bromovinyl)-2-methoxyphenol (**8b**) in GC-MS.

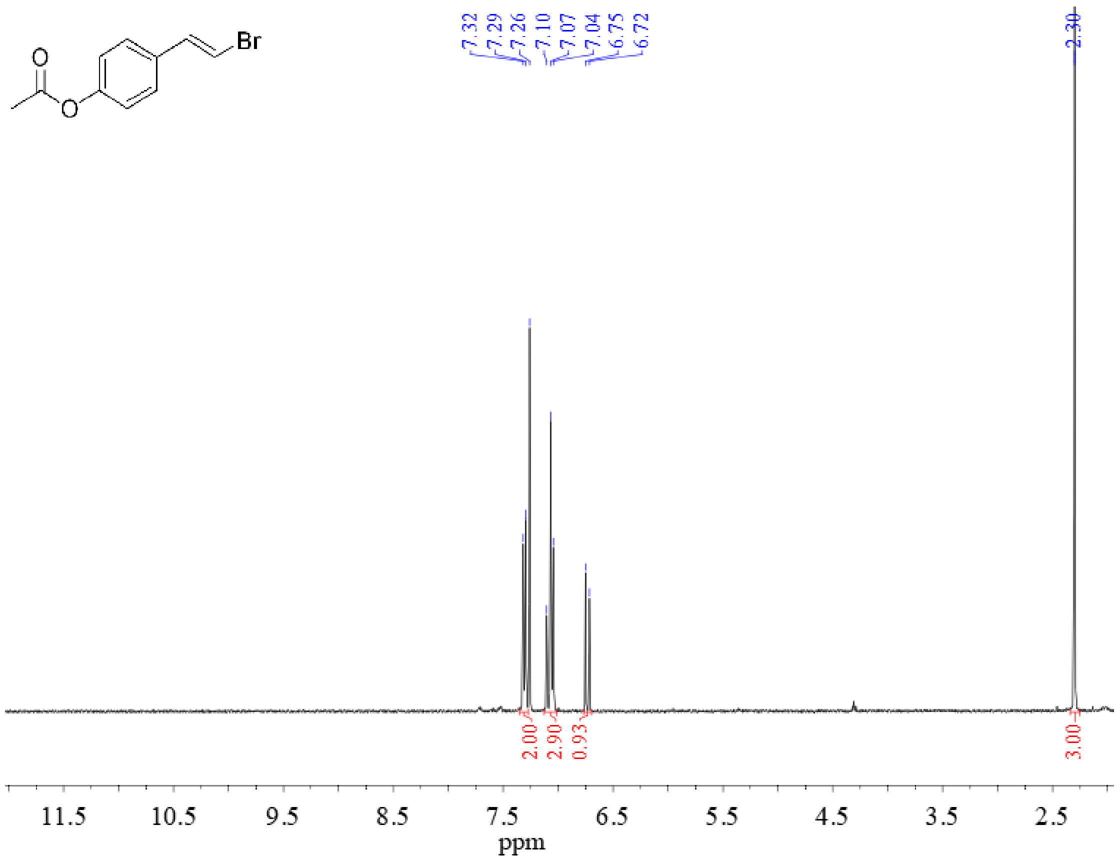

Figure S26. ^{1}H NMR spectrum of 4-(2-bromovinyl)-phenyl acetate (**9b**) in $CDCl_3$.

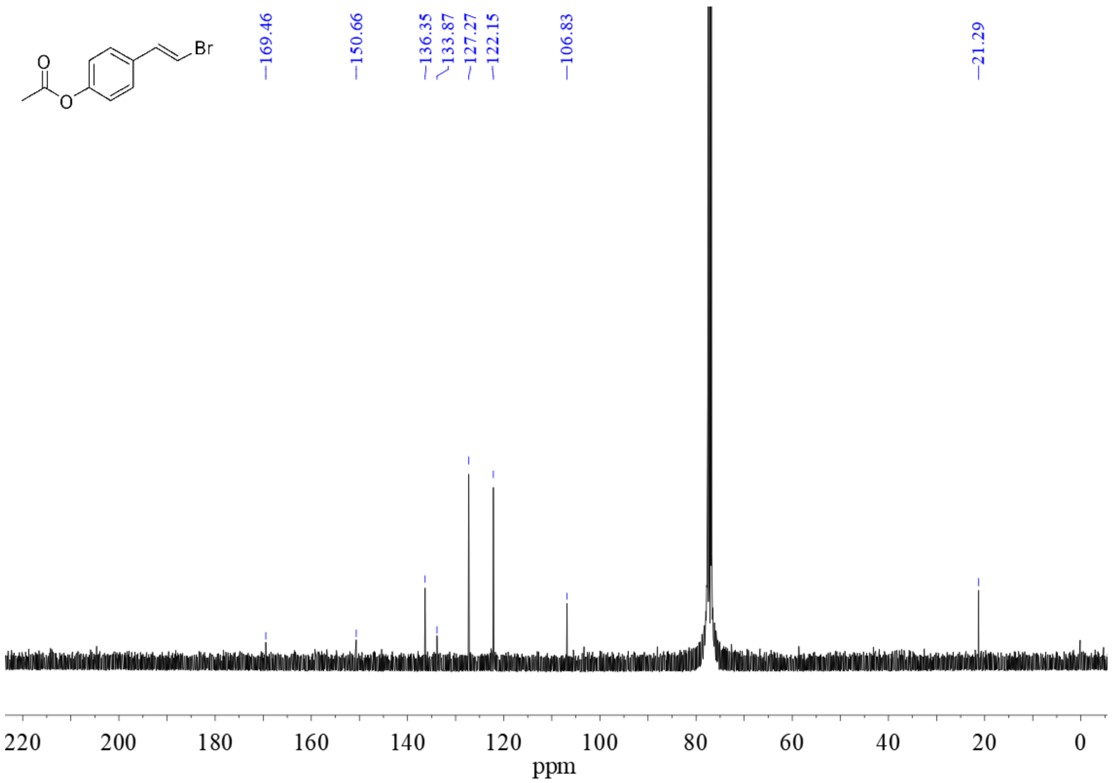

Figure S27. ^{13}C NMR spectrum of 4-(2-bromovinyl)-phenyl acetate (**9b**) in $CDCl_3$.

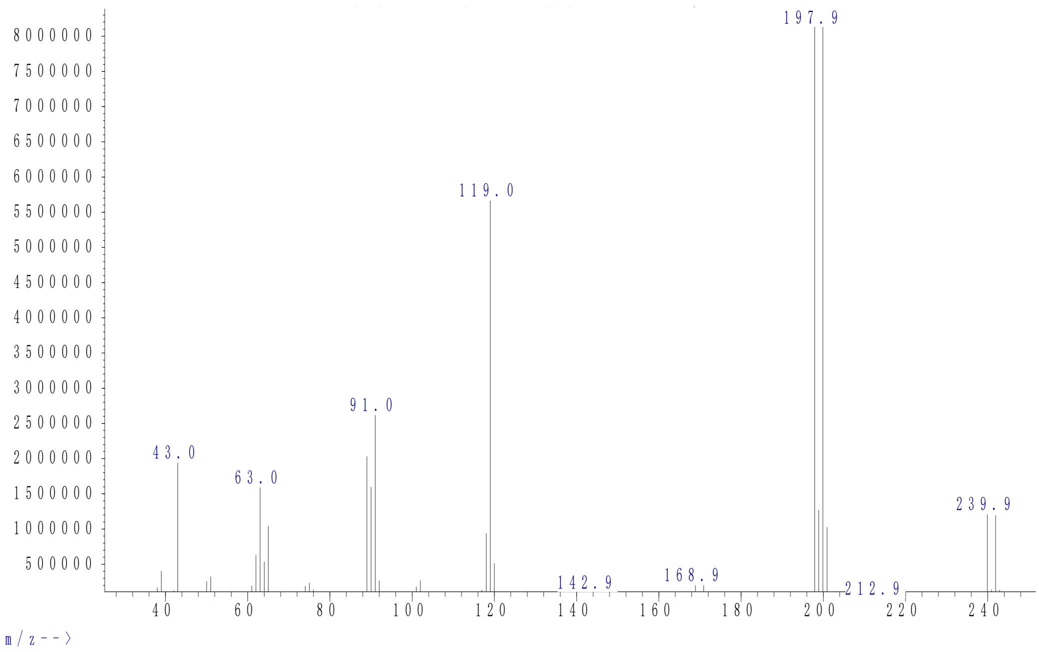

Figure S28. Mass spectrum of (E)-4-(2-bromovinyl)-phenyl acetate (**9b**) in GC-MS.

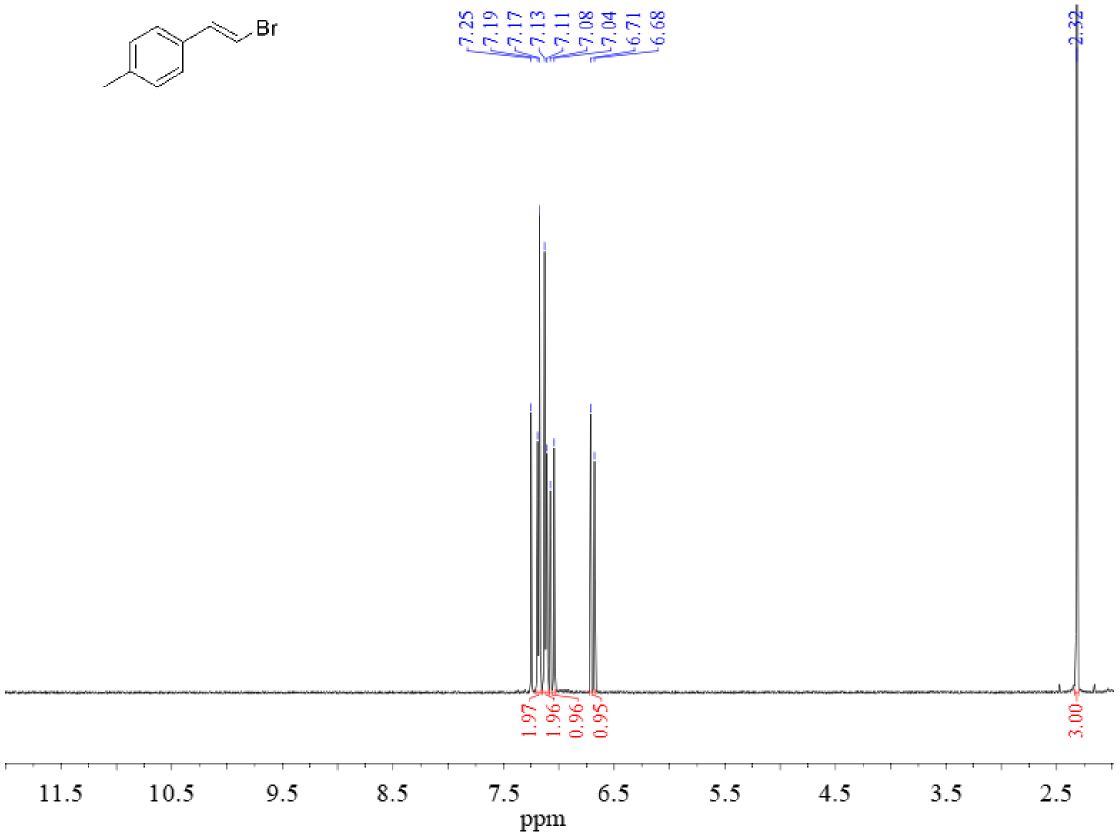

Figure S29. ^{1}H NMR spectrum of 1-(2-bromovinyl)-4-methylbenzene (**10b**) in $CDCl_3$.

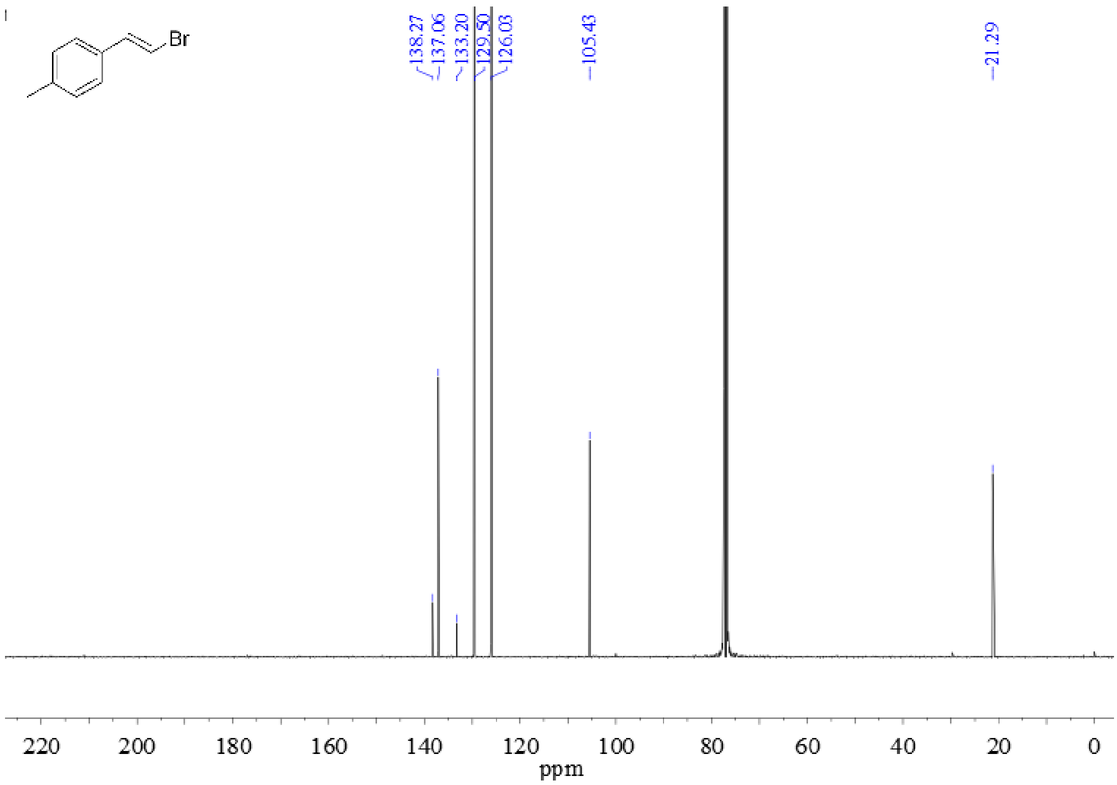

Figure S30. ^{13}C NMR spectrum of 1-(2-bromovinyl)-4-methylbenzene (**10b**) in $CDCl_3$.

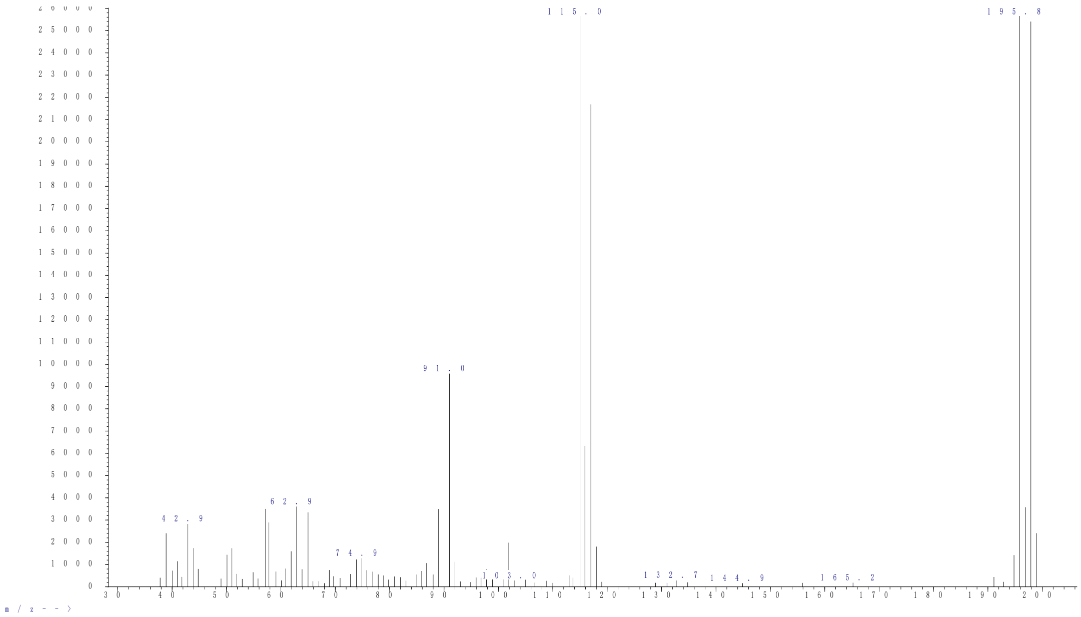

Figure S31. Mass spectrum of 1-(2-bromovinyl)-4-methylbenzene (**10b**) in GC-MS.

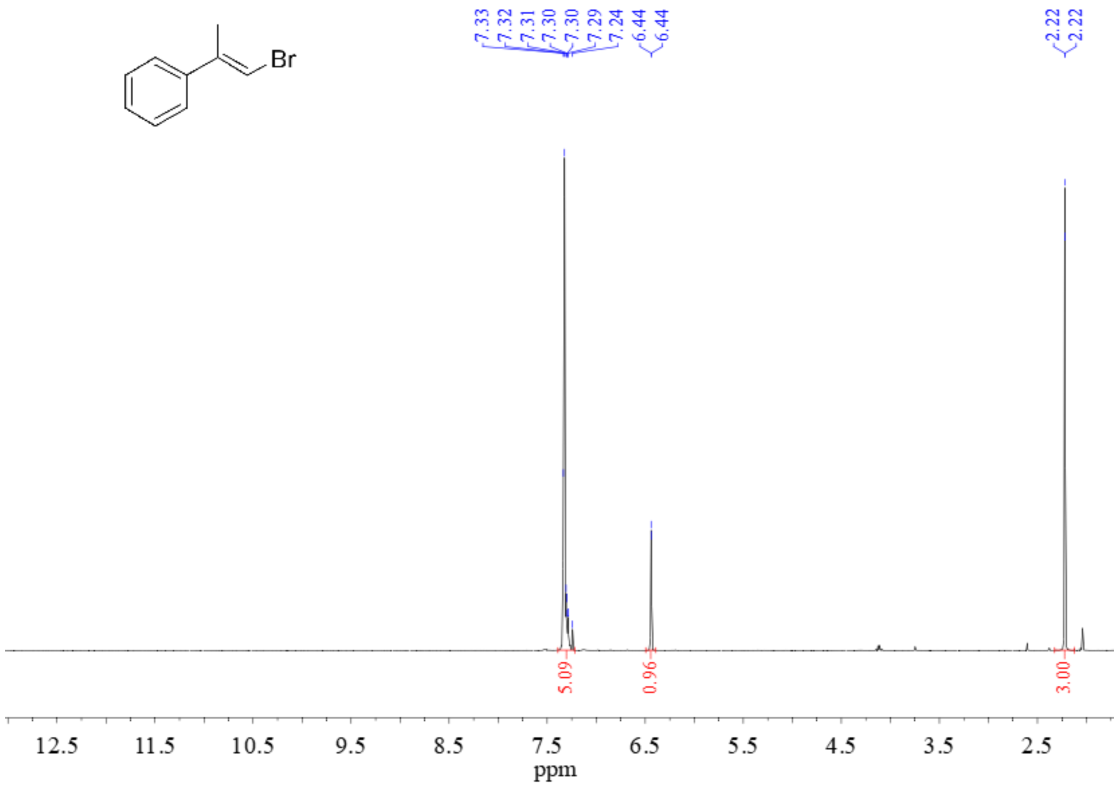

Figure S32. ^{1}H NMR spectrum of 1-bromo-2-phenylpropene (**11b**) in $CDCl_3$.

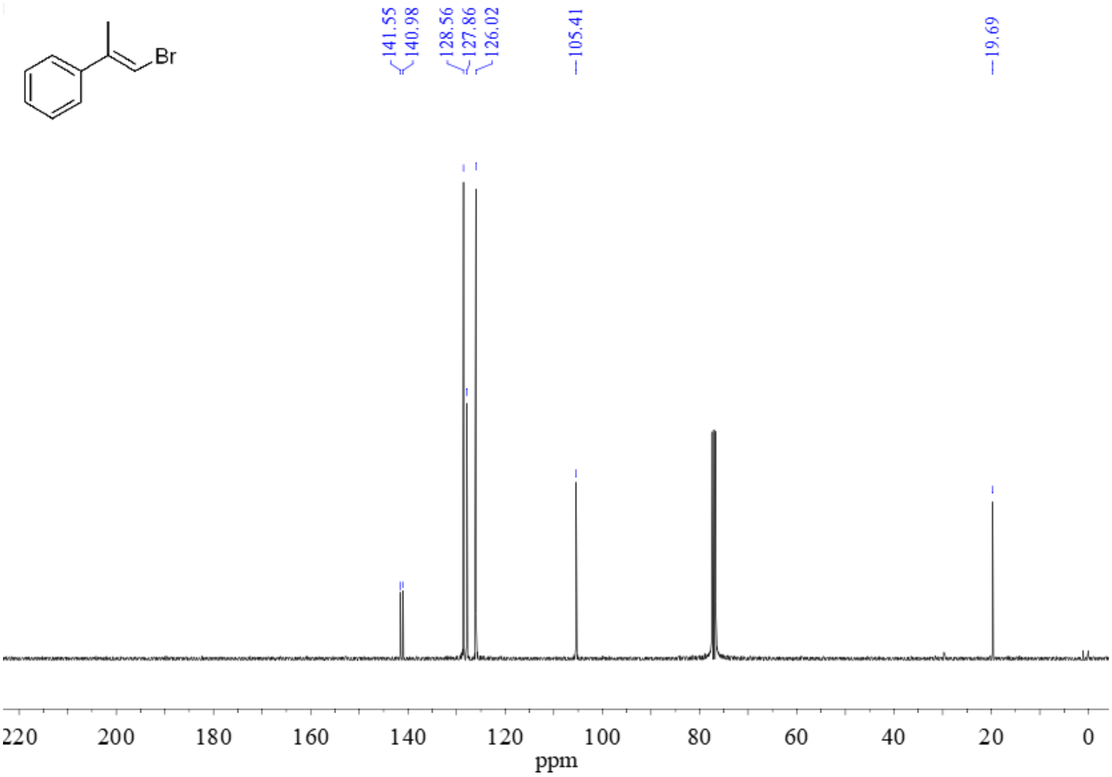

Figure S33. ^{13}C NMR spectrum of 1-bromo-2-phenylpropene (**11b**) in $CDCl_3$.

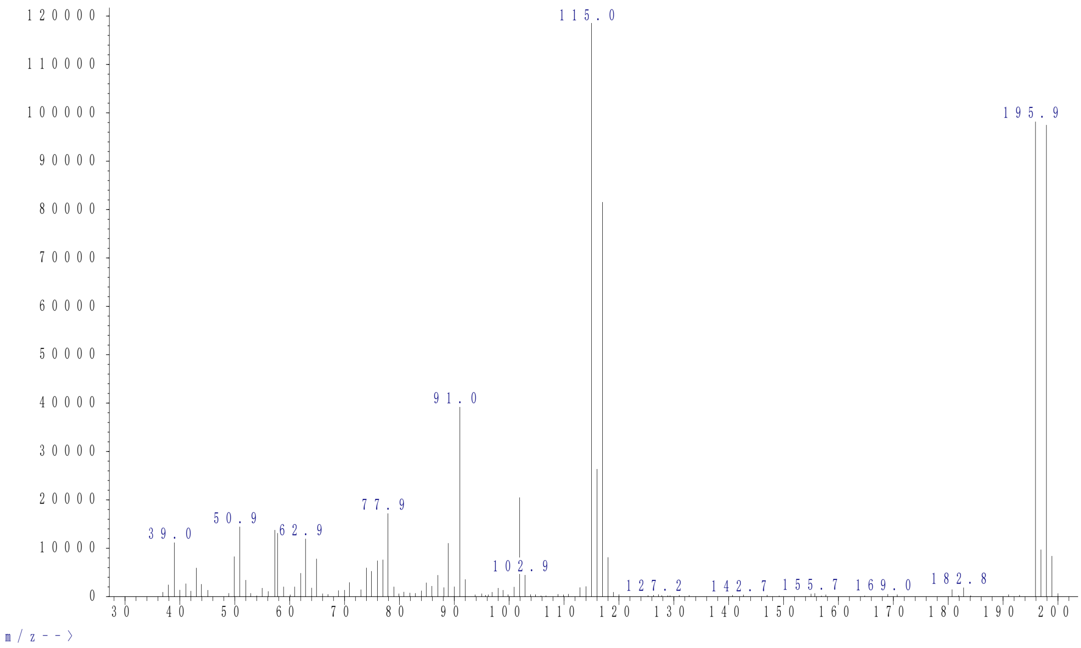

Figure S34. Mass spectrum of 1-bromo-2-phenylpropene (**11b**) in GC-MS.

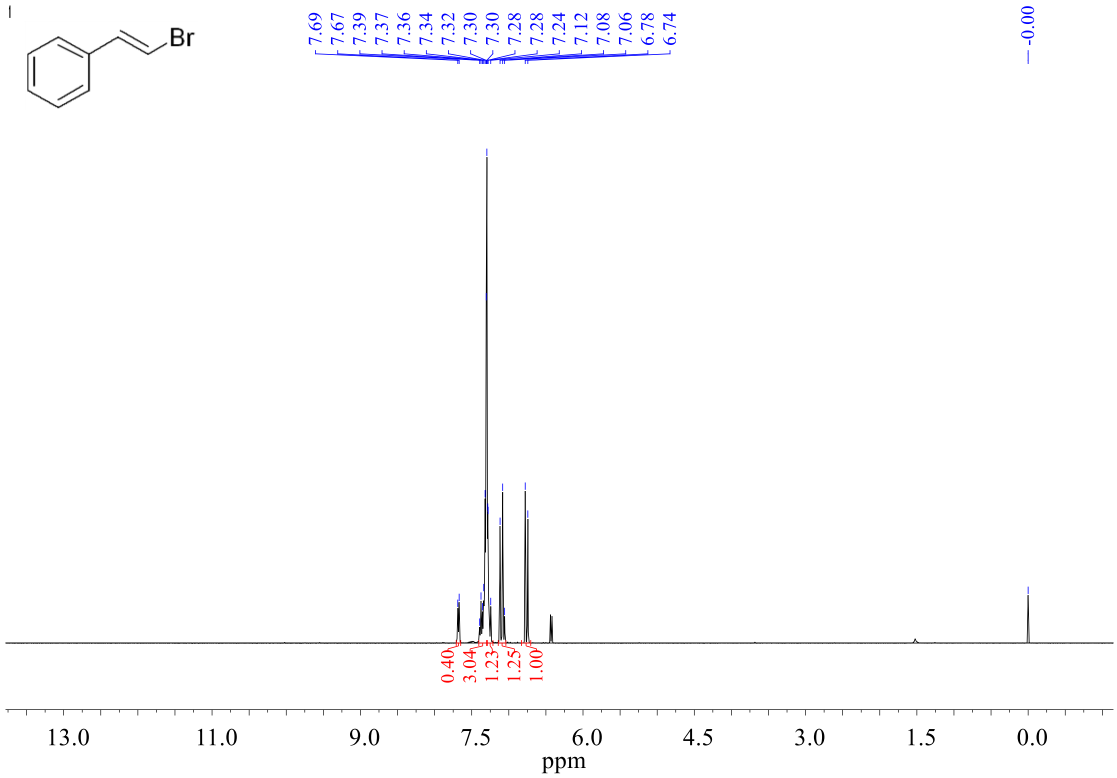

Figure S35. ^{1}H NMR spectrum of (2-bromovinyl) benzene (**12b**) in $CDCl_3$.

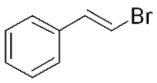

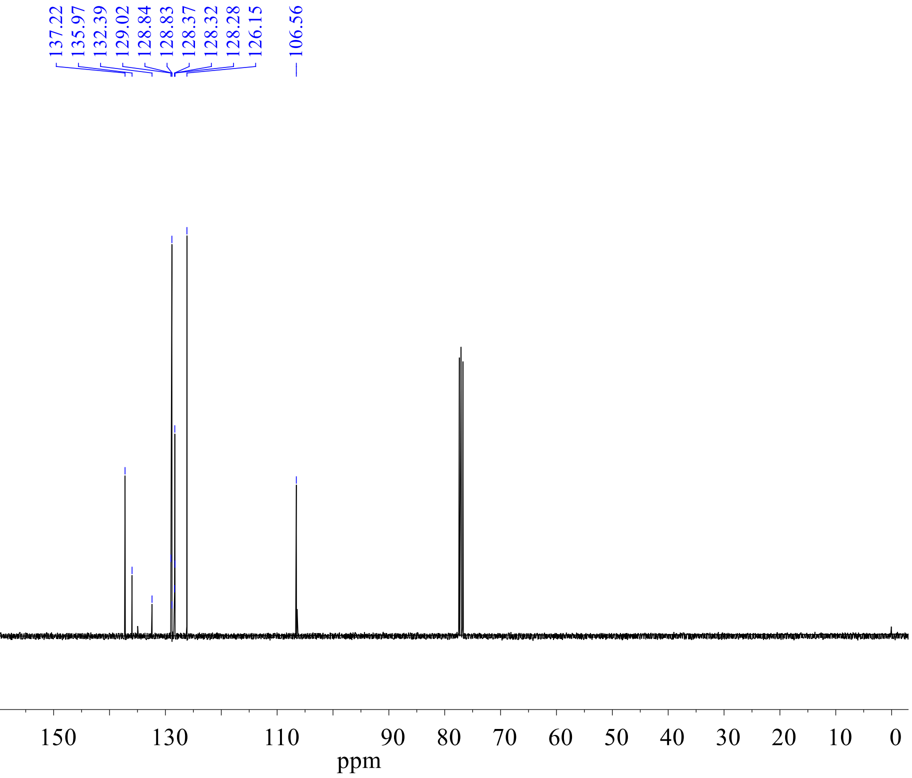

Figure S36. ^{13}C NMR spectrum of (2-bromovinyl) benzene (**12b**) in $CDCl_3$.

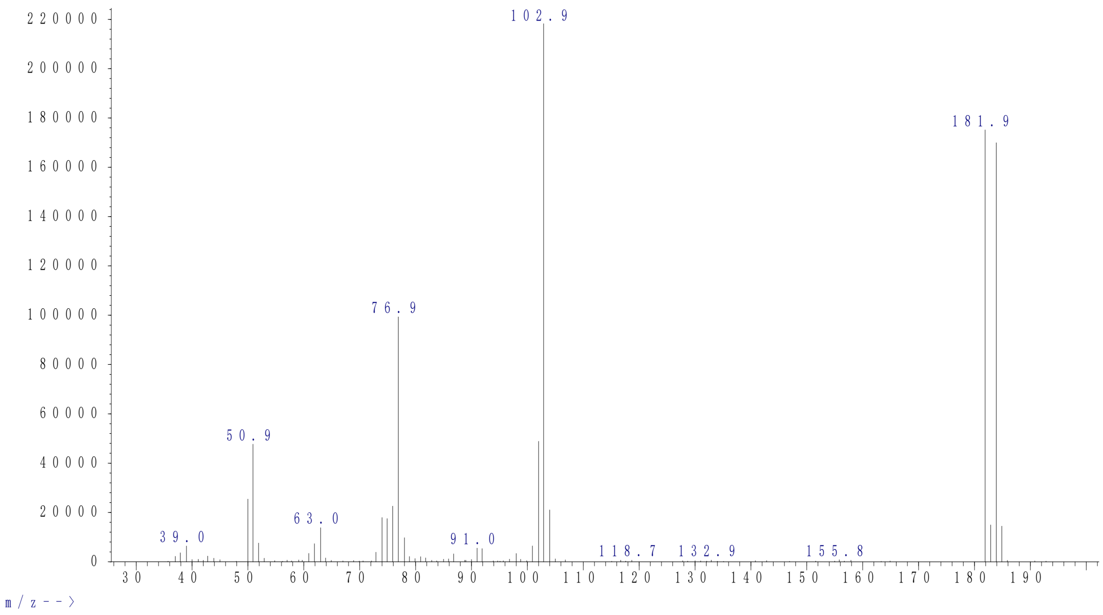

Figure S37. GC-MS spectrum of (2-bromovinyl) benzene (**12b**).

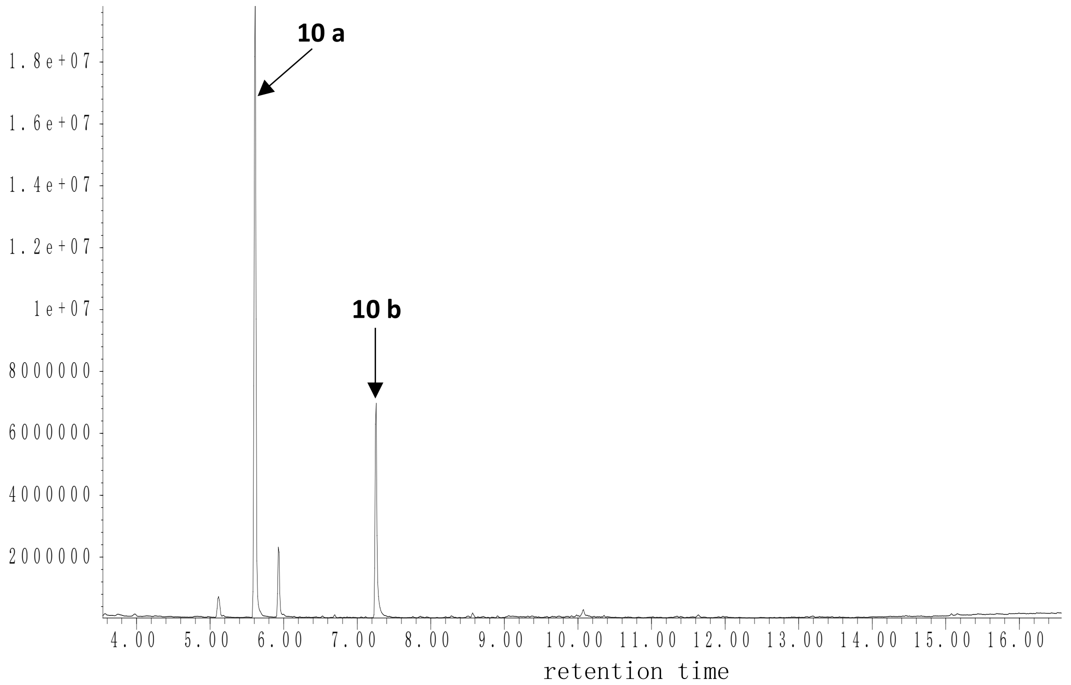

Figure S38. Representative GC-MS chromatogram of the reaction mixture of the decarboxylation of 3-(p-tolyl) acrylic acid (**10a**) with 5% DMSO in 1mL scale.

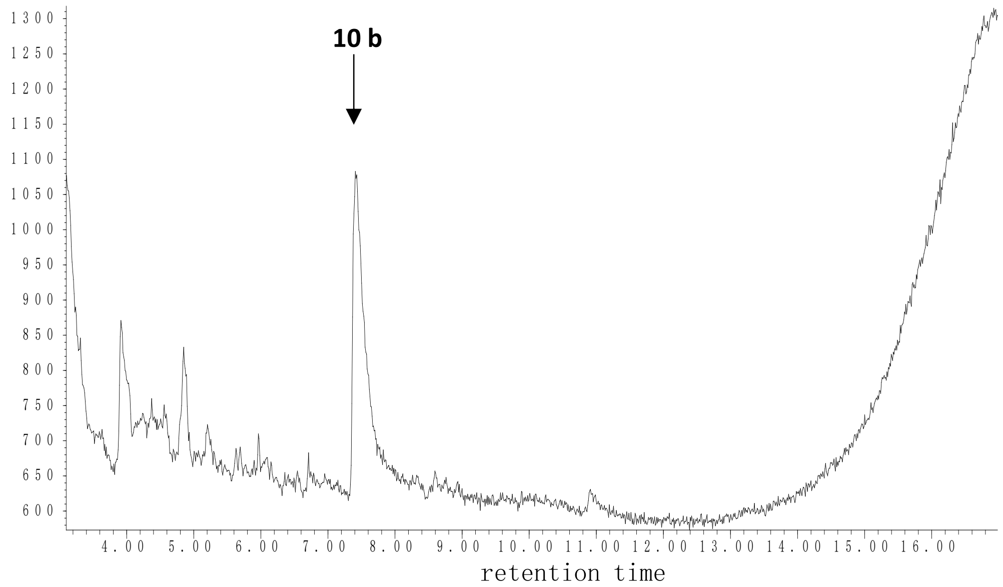

Figure S39. Representative GC-MS chromatogram of the reaction mixture of the decarboxylation of 3-(p-tolyl) acrylic acid (**10a**) with 50% DMSO in 1mL scale.

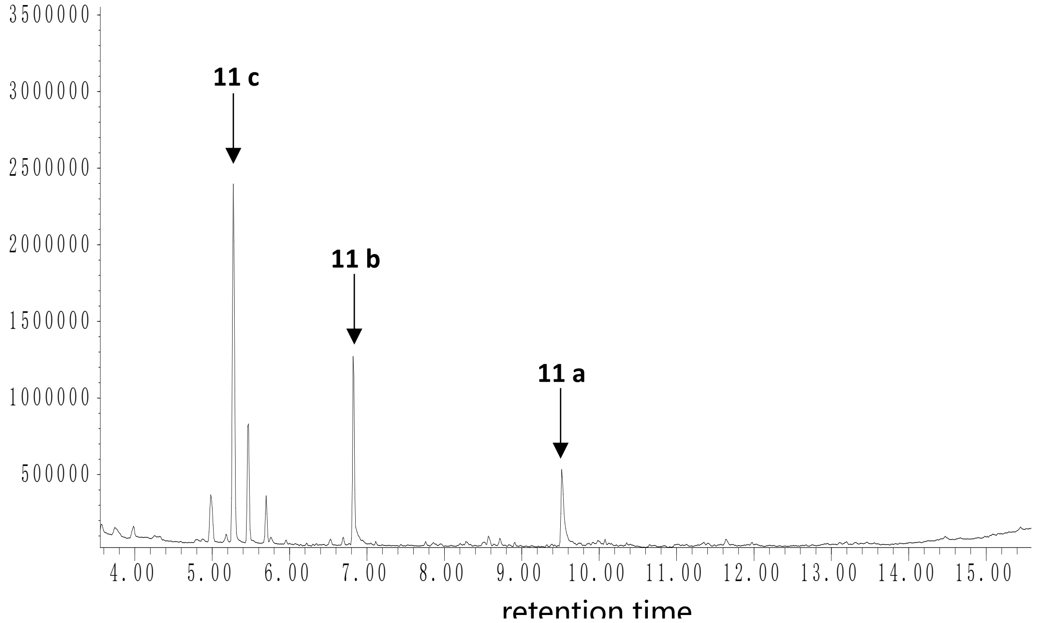

Figure S40. Representative GC-MS chromatogram of the reaction mixture of the decarboxylation of 3-phenylbut-2-enoic acid (**11a**) with 5% DMSO in 1mL scale.

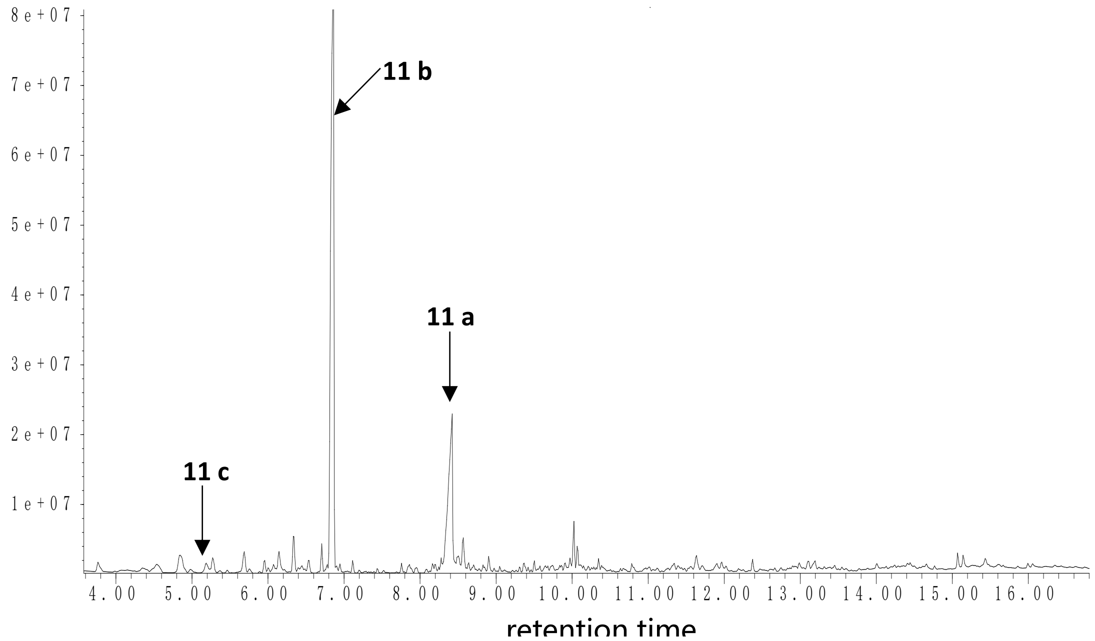

Figure S41. Representative GC-MS chromatogram of the reaction mixture of the decarboxylation of 3-phenylbut-2-enoic acid (**11a**) with 50% DMSO in 1mL scale.

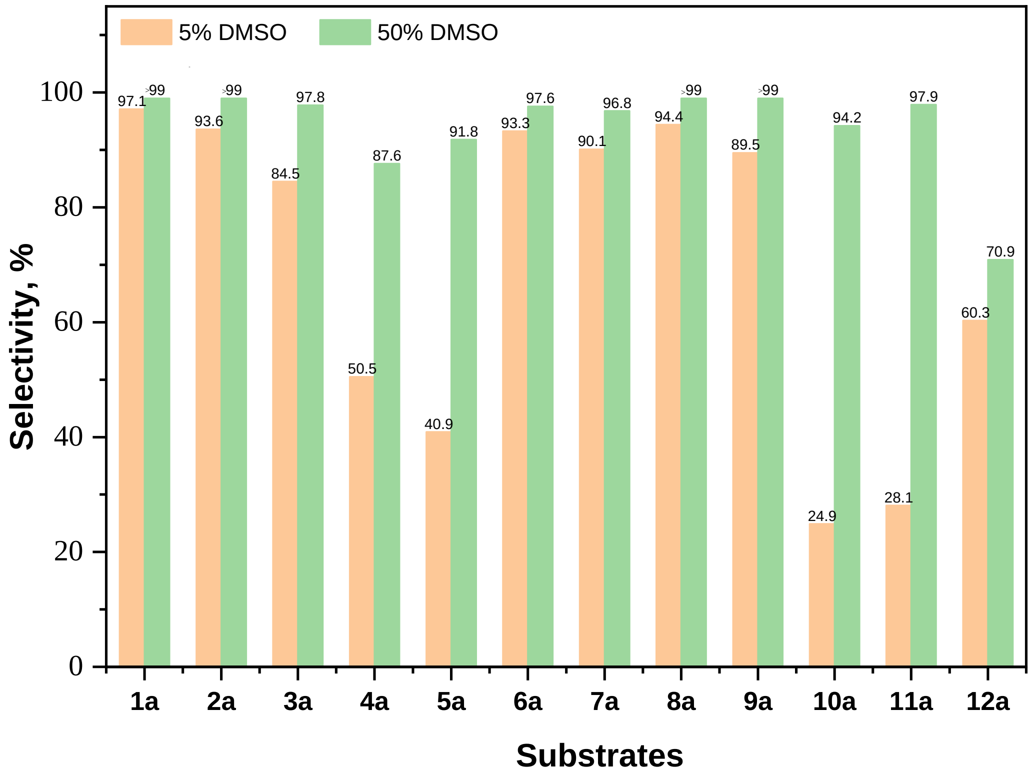

Figure S42. Comparison of the selectivity of the conversions of starting materials **1a-12a** in the presence of either 5% or 50% DMSO in 1mL scale.

Synthesis of TXT catalyst

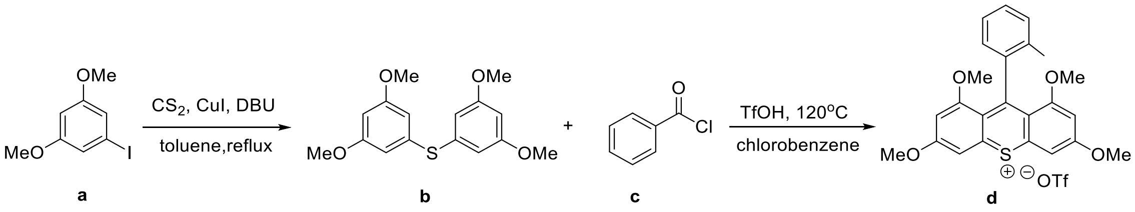

The catalyst TXT was synthesized according to previously reported methods[2].

Step1: The synthesis of bis(3,5-dimethoxyphenyl) sulfane

A mixture of iodoarene **a** (600mg), carbon disulfide (140ul), CuI (0.044g), and DBU (680 ul) in toluene (8mL) were stirred in 25mL three-necked bottle at reflux under N_2 for 12 h. The reaction was monitored by using TLC. Upon the completion of the reaction, H_2O was added and then the solution was extracted with CH_2Cl_2. The organic layer was combined and dried over Na_2SO_4 and concentrated in vacuo. The crude mixture was purified by column chromatography on silica gel (petroleum/ethyl acetate = 85% / 15%) to provide the corresponding bis(3,5-dimethoxyphenyl) sulfane **b** as a white solid.

Step2: The synthesis of 9-(2-Methylphenyl)-1,3,6,8-tetramethoxythioxanthylium trifluoromethanesulfonate (TXT)

To a solution of thioether **b** (150mg) and benzoyl chloride (232mg) in chlorobenzene (5.0 mL) was stirred in a 25 mL flask under N_2. Then the trifluoromethanesulfonic acid (400ul) was slowly added to the solution and the mixture was heated to 120 °C and kept for 1 h. After cooling to room temperature excess Et_2O was added to precipitate the target compound as a solid. The solid was filtered and thoroughly washed with Et_2O and dried in vacuo, affording the desired thioxanthylium product **d** as a brown solid (TXT).

Bis(3,5-dimethoxyphenyl) sulfane (b)

White solid (150mg, 43.0% yield)

^{1}H NMR (400 MHz, $CDCl_3$): δ 6.84 (d, J = 2.2 Hz, 1H), 6.51 (d, J = 2.2 Hz, 3H), 6.34 (dd, J = 2.9, 1.5 Hz, 2H), 3.73 (s, 12H).

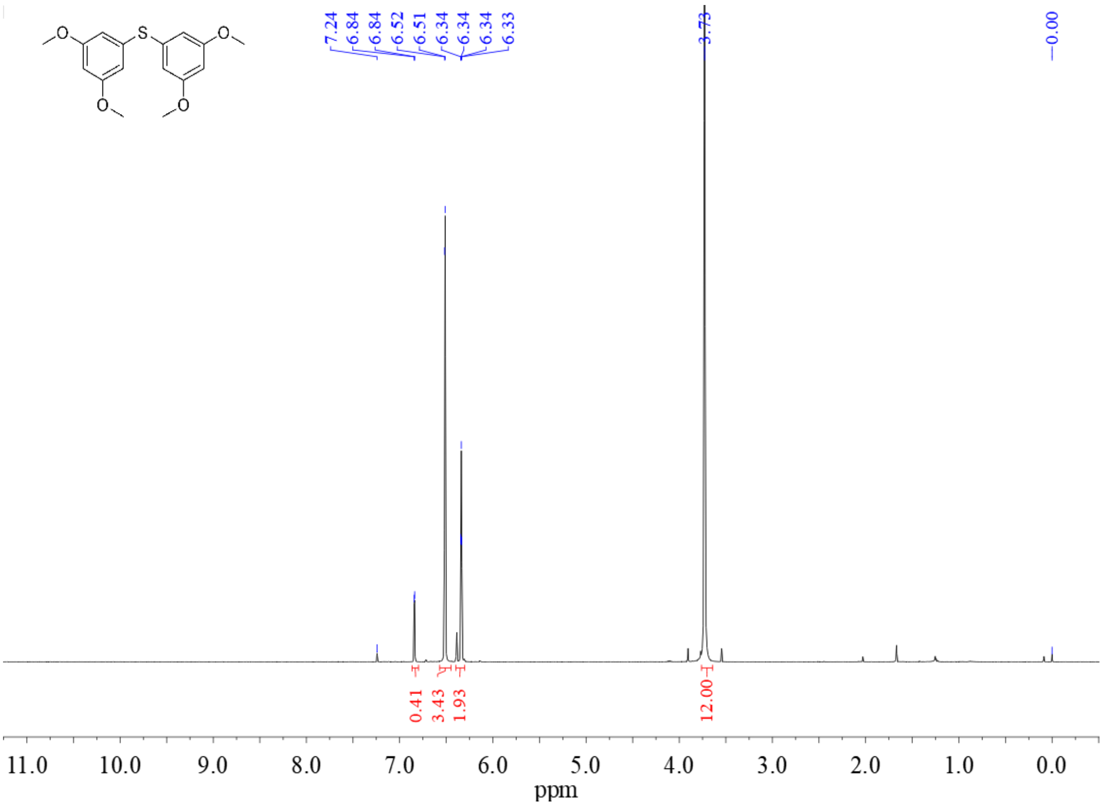

Figure S43. ^{1}H NMR spectrum of Bis(3,5-dimethoxyphenyl) sulfane (**b**) in $CDCl_3$.

9-(2-Methylphenyl)-1,3,6,8-tetramethoxythioxanthylium trifluoromethanesulfonate (TXT, d)

Brown solid (66.8mg, 24.5% yield)

^{1}H NMR (400 MHz, $CDCl_3$): δ 7.56 (s, 2H), 7.30 (d, *J* = 7.5 Hz, 1H), 7.25 – 7.16 (m, 2H), 6.74 (d, *J* = 7.2 Hz, 1H), 6.52 (s, 2H), 4.16 (s, 6H), 3.39 (s, 6H), 2.03 (s, 3H).

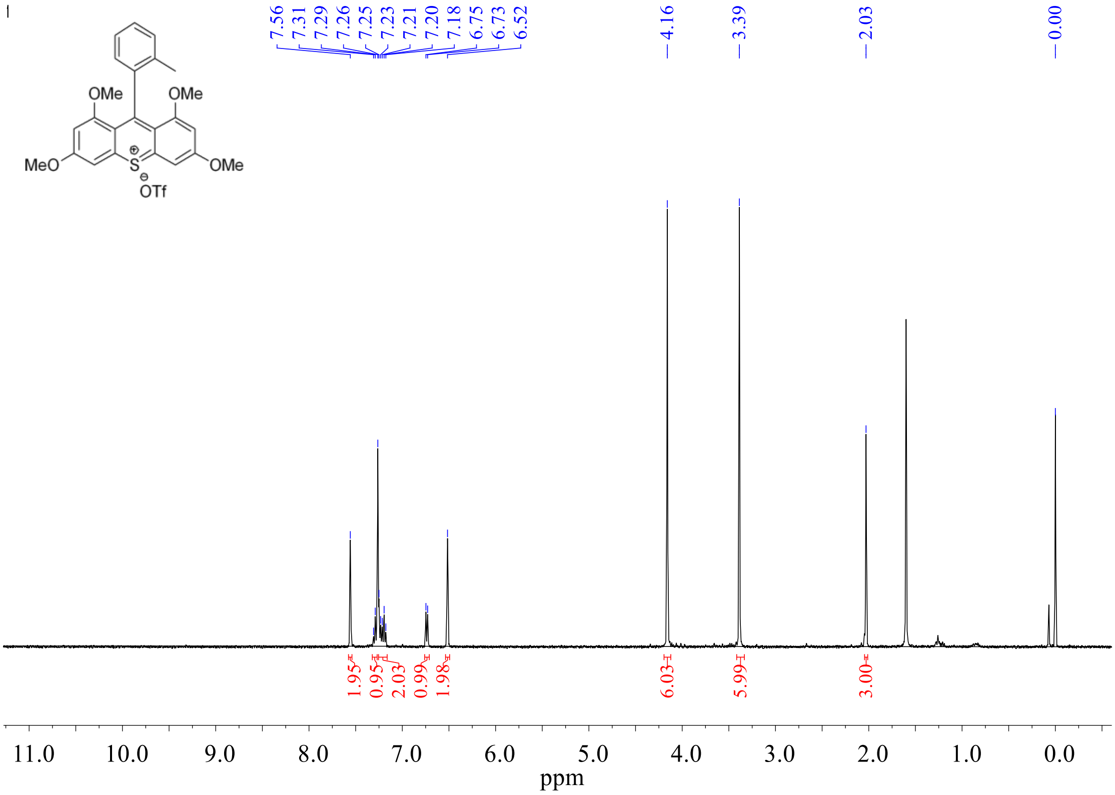

Figure S44. ^{1}H NMR spectrum of **TXT** photocatalyst in $CDCl_3$.

General procedure for crossed intermolecular cyclic [2+2] cycloaddition

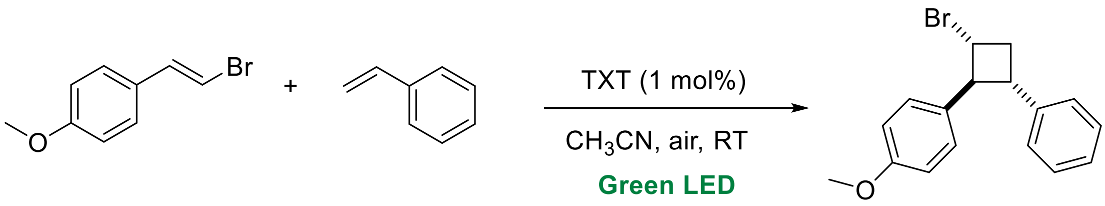

To a 25 mL round bottom flask containing 8mL CH_3CN, **3b** (0.22mmol), styrene (2.2mmol) and 3 mol% TXT photocatalyst were added. The mixture was stirred and irradiated using a green LED (24W) at room temperature for 24h. After the reaction, the solvent was evaporated under reduced pressure. In a final step, the reaction mixture was purified by flash chromatography using 10% ethyl acetate in petroleum ether to obtain the desired product as a white solid.

1-((1S,2R,4S)-2-bromo-4-phenylcyclobutyl)-4-methoxybenzene

white solid (24.3mg, 35%yield)

^{1}H NMR (400 MHz, $CDCl_3$): δ 7.33 – 7.28 (m, 2H), 7.25 – 7.18 (m, 5H), 6.86 (m,2H), 4.35 (dd, 1H), 3.82-3.75 (m, 4H), 3.42 (d, 1H), 3.02 (1H), 2.57 (dd, 1H).

^{13}C NMR (101 MHz, $CDCl_3$): δ 158.86 (s), 142.07 (s), 132.36 (s), 128.63 (s), 127.60 (s), 126.70 (d, J = 17.1 Hz), 114.09 (s), 59.56 (s), 55.33 (s), 44.39 (d, J = 1.7 Hz), 38.64 (s).

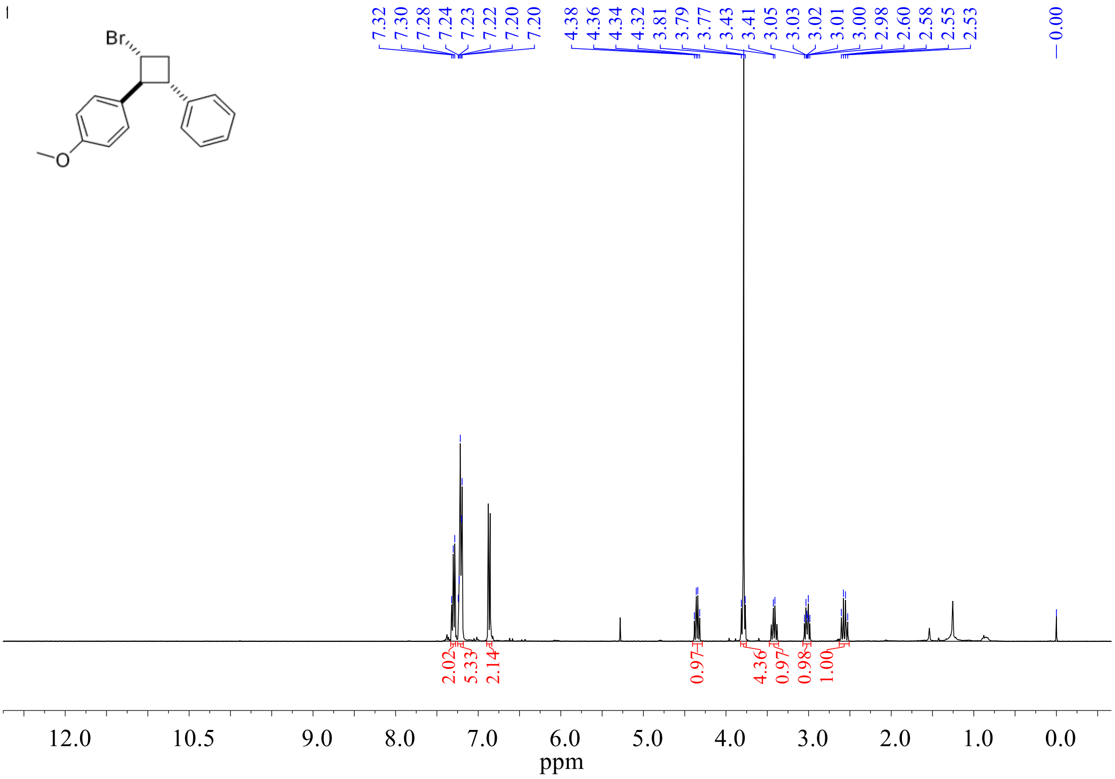

Figure S45. ^{1}H NMR spectrum of 1-((1S,2R,4S)-2-bromo-4-phenylcyclobutyl)-4-methoxybenzene in $CDCl_3$.

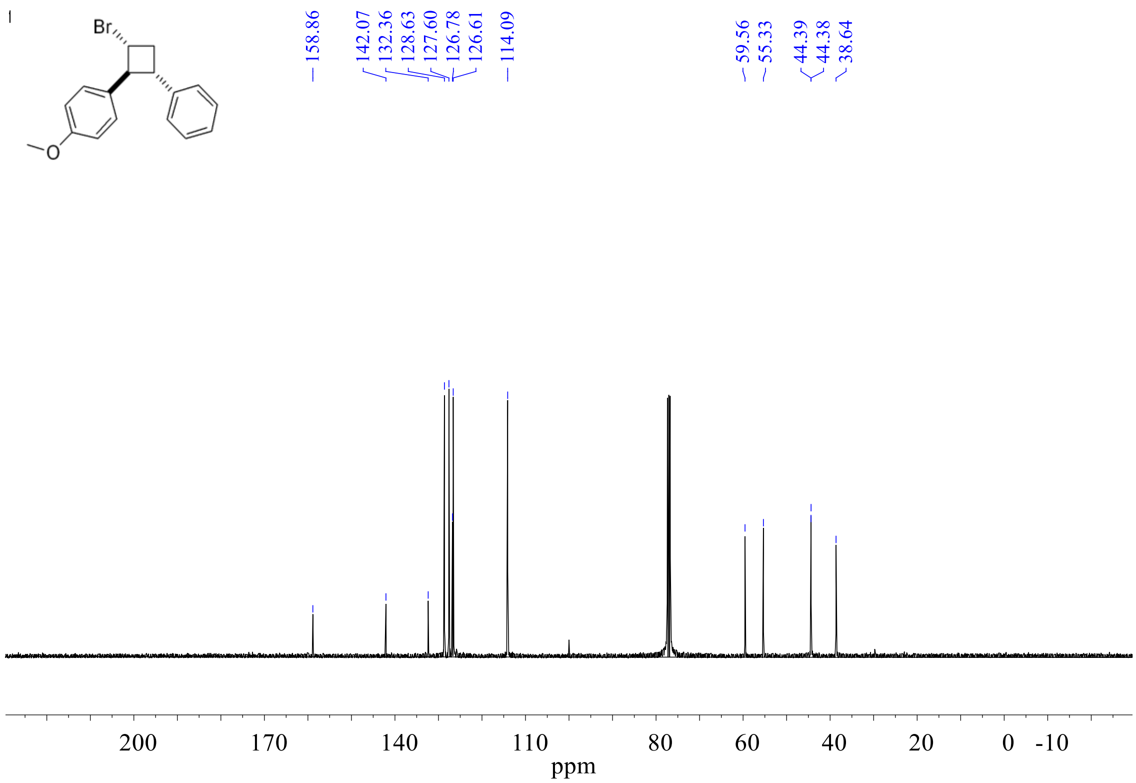

Figure S46. ^{13}C NMR spectrum of 1-((1S,2R,4S)-2-bromo-4-phenylcyclobutyl)-4-methoxybenzene in $CDCl_3$.

General procedure for the Suzuki–Miyaura cross-coupling reaction

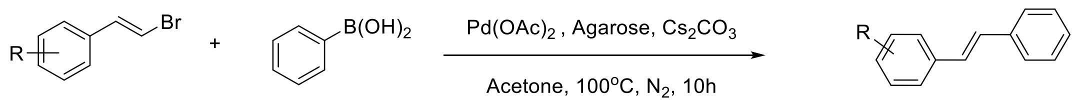

3b (0.1mmol) or **12b** (0.3mmol), phenylboronic acid (0.12-0.36mmol), $Pd(OAc)_2$ (3mol%), agarose (6mol%), and CS_2CO_3 (0.5mmol) in 5mL acetone at 100 °C in nitrogen were stirred in a Schlenk tube for about 16h. After the reaction, the mixture was poured into ethyl acetate, washed with brine, and extracted with ethyl acetate. The combined organic layers were evaporated in vacuo. The residue was purified by flash column chromatography using 1% ethyl acetate in petroleum ether to obtain the desired product as a white solid.

1-methoxy-4-styrylbenzene

White solid (12.6mg, 60% yield)

^{1}H NMR (400 MHz, CDCl$_3$): δ 7.47 (dd, *J* = 13.2, 8.1 Hz, 4H), 7.34 (t, *J* = 7.6 Hz, 2H), 7.23 (s, 1H), 7.07 (d, *J* = 16.4 Hz, 1H), 6.97 (d, *J* = 16.3 Hz, 1H), 6.90 (d, *J* = 8.7 Hz, 2H), 3.83 (s, 3H).
^{13}C NMR (101 MHz, CDCl$_3$): δ 159.34 (s), 137.69 (s), 130.19 (s), 128.68 (s), 128.24 (s), 127.75 (s), 127.25 (s), 126.66 (s), 126.28 (s), 114.17 (s), 100.00 (s), 55.37 (s).

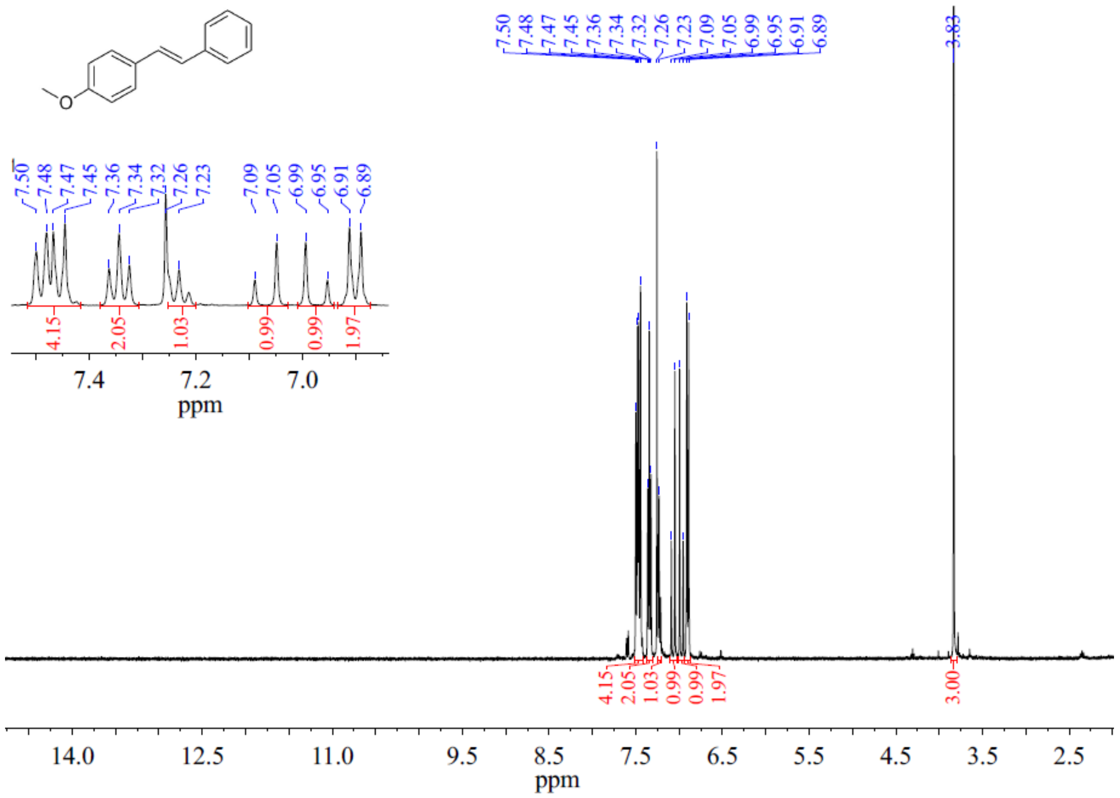

Figure S47. ^{1}H NMR spectrum of 1-methoxy-4-styrylbenzene in CDCl$_3$.

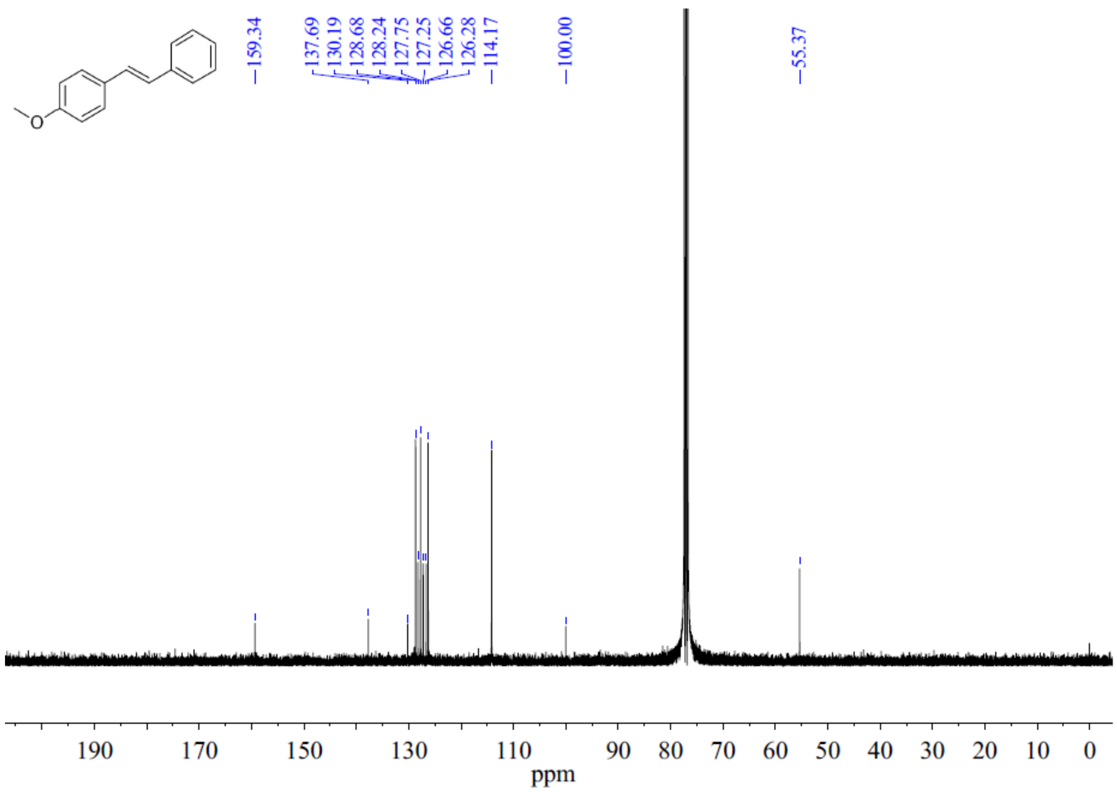

Figure S48. ^{13}C NMR spectrum of 1-methoxy-4-styrylbenzene in $CDCl_3$.

1,2-Diphenylethene

White solid (35.1mg, 65% yield)

^{1}H NMR (400 MHz, CDCl$_3$): δ 7.52 (d, *J* = 7.4 Hz, 4H), 7.36 (t, *J* = 7.7 Hz, 5H), 7.28 (s, 1H), 7.24 (d, *J* = 5.1 Hz, 1H), 7.11 (s, 2H).

^{13}C NMR (101 MHz, CDCl$_3$): δ 137.39 (s), 128.75 (d, *J* = 1.5 Hz), 127.68 (s), 126.58 (s).

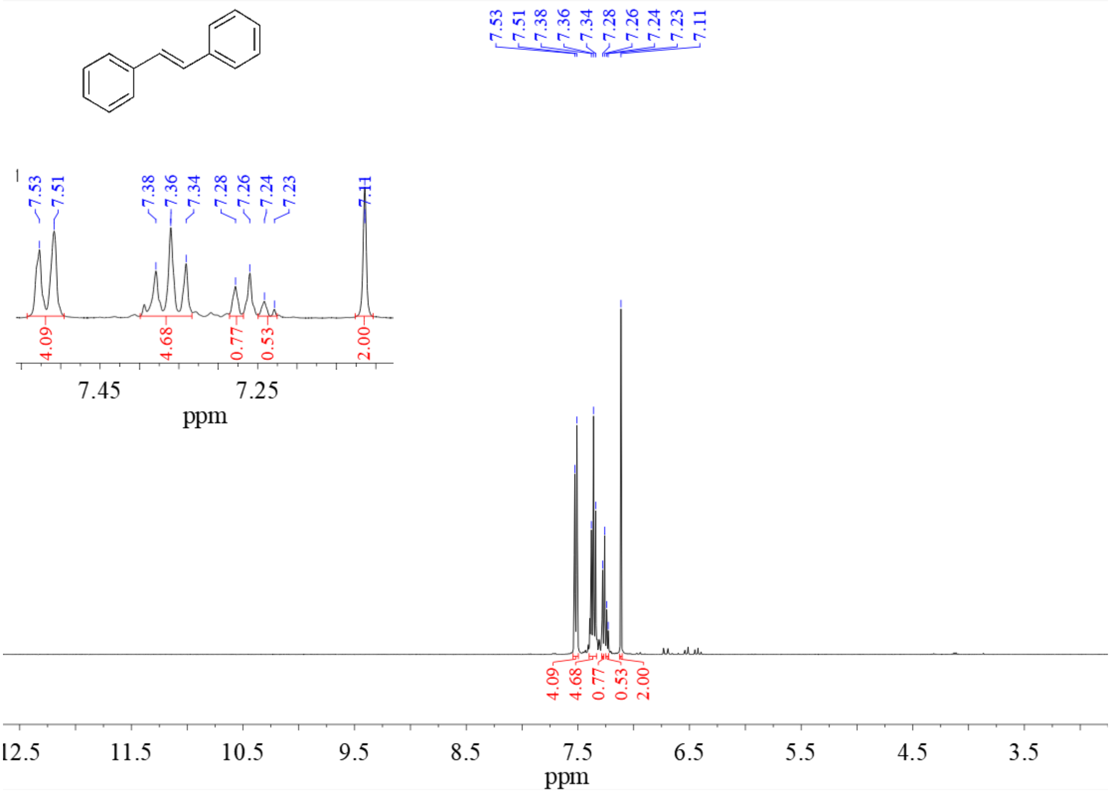

Figure S49. ^{1}H NMR spectrum of 1,2-diphenylethene in CDCl$_3$.

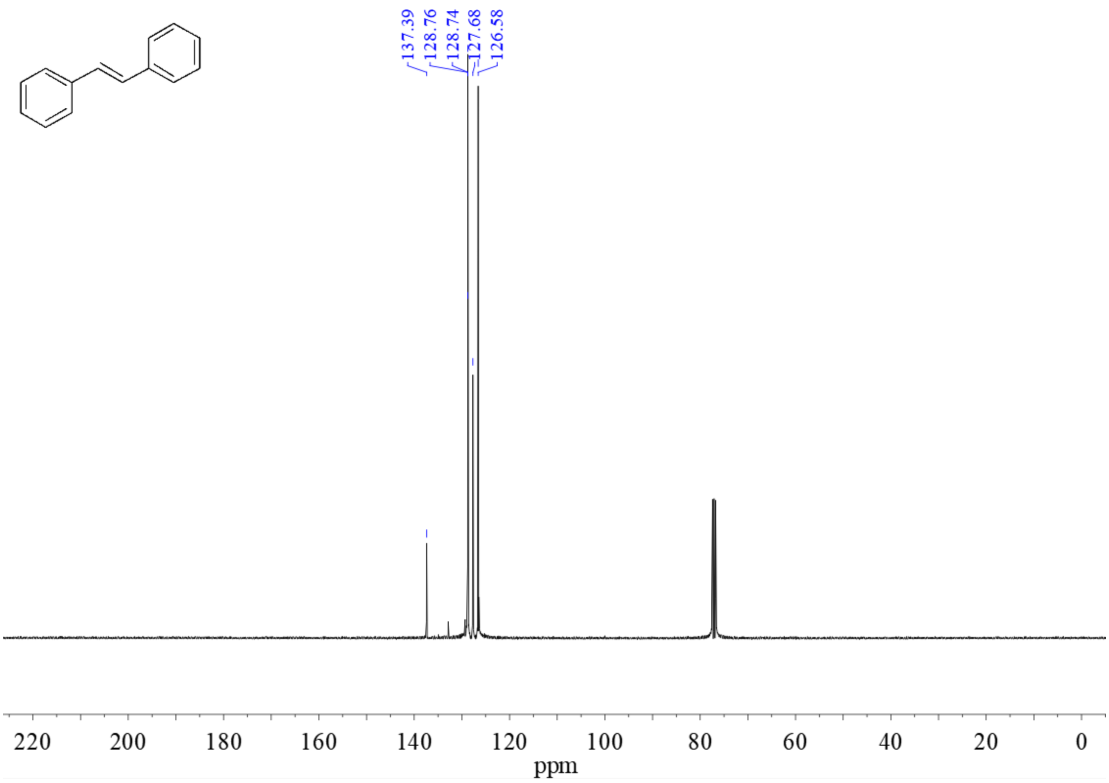

Figure S50. ^{13}C NMR spectrum of 1,2-diphenylethene in $CDCl_3$.

General procedure for the homo-coupling reaction

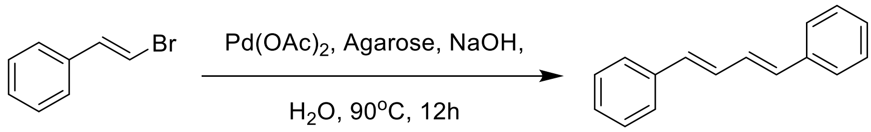

Agarose (0.05g) and Pd (OAc)$_2$ (0.02mol) were prepared in a water-containing (2 mL) flack (10mL) and heated at 90 ^oC with stirring for 5 min. Then, the β-bromo styrene (**12b**) (1mmol) and NaOH (1.5 mmol) were added and stirred at 90 ^oC under the air for 12 h. After the reaction, the mixture was cooled and the resulting mixture was extracted with acetic ether, and dried under reduced pressure to obtain the corresponding crude product. The residue was purified by flash column chromatography (pure petroleum) providing the desired homo-coupling product.

1,4-Diphenylbuta-1,3-diene

White solid (65.9mg, 64%yield)

1H NMR (400 MHz, CDCl3): δ 7.49 (d, 4H), 7.40-7.36 (t, 4H), 7.30 -7.26 (d, 3H), 7.06 – 6.96 (m, 2H), 6.79 – 6.69 (m, 2H).

^{13}C NMR (101 MHz, $CDCl_3$): δ 137.39 (s), 132.85 (s), 129.28 (s), 128.70 (s), 127.60 (s), 126.42 (s).

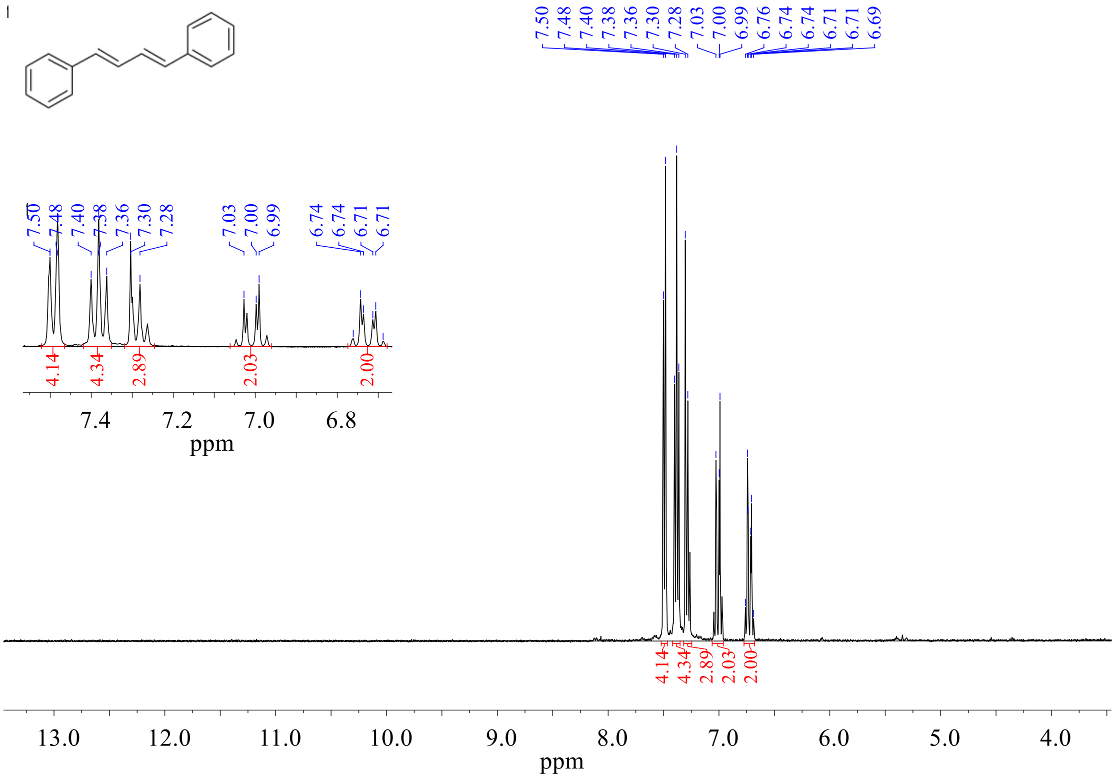

Figure S51. ^{1}H NMR spectrum of 1,4-diphenylbuta-1,3-diene in $CDCl_3$.

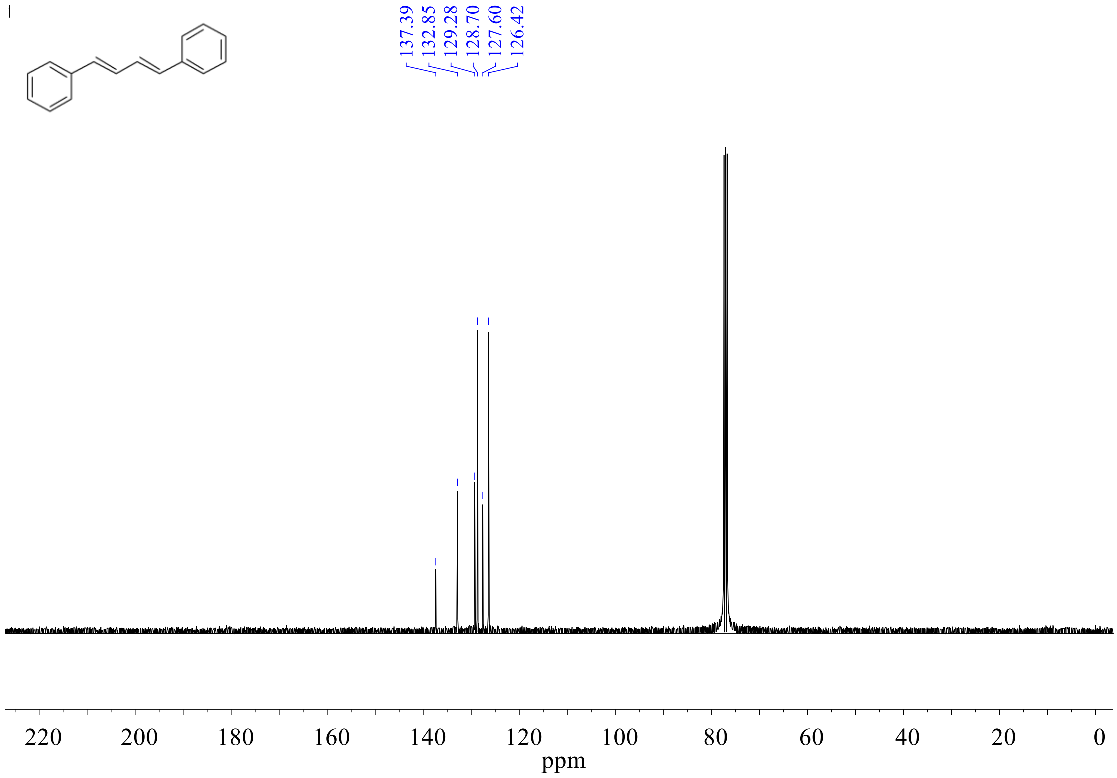

Figure S52. ^{1}H NMR spectrum of 1,4-diphenylbuta-1,3-diene in $CDCl_3$.

Table S1. Control reaction for the bromination reaction of *p*-coumaric acid.

Entry	Catalyst		pH	c(H_2O_2) (mM)	Concentration (mM)
	Inorg. Cat.	c(*Ci*VCPO) (nM)			
1	-	-	5	30	N.D.
2	-	400[a]	5	30	N.D.
3[b]	-	400	5	-	N.D.
4[c]	Na_3VO_4	-	5	30	N.D.
5[d]	NaClO	-	5	-	N.D.

Reaction conditions unless specified: [p-coumaric acid] = 30 mM, citrate buffer (100 mM, pH 5.0), [*Ci*VCPO]= 400 nM, [KBr] = 50 mM, [H_2O_2] = 30 mM, 30 °C, 5% DMSO, 6 h, 1 mL. [a] *Ci*VPCO was boiled in 100 °C water for 3h to inactivate. [b] No H_2O_2 was added. [c] 400 nM or 10 uM Na_3VO_4 was added. [d] 50 mM NaClO was added to the reaction system at a rate of 6 mM h^{-1}. N.D. means not detected.

Table S2. NMR data of the obtained vinyl halides products (**1b-12b**)

Compound	NMR data
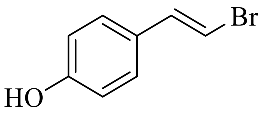<br>4-(2-bromovinyl) phenol (**1b**)<br>White solid (172.8mg, 58.2% yield)	**^{1}H NMR (400 MHz, CDCl$_3$):** δ 7.22 –7.16 (m, 2H), 7.03 (d, *J* = 13.9 Hz, 1H), 6.81 – 6.76 (m, 2H), 6.60 (d, *J* = 13.9 Hz, 1H), 5.12 (s, 1H).<br><br>**^{13}C NMR (101 MHz, CDCl$_3$):** δ 155.59 (s), 136.45 (s), 129.04 (s), 127.58 (s), 115.68 (s), 104.15 (s).
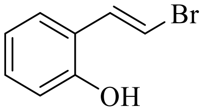<br>2-(2-bromovinyl) phenol (**2b**)<br>Grey white solid (223.9mg, 75.4% yield)	**^{1}H NMR (400 MHz, CDCl$_3$):** δ 7.29 (s, 1H), 7.24 (dd, *J* = 7.7, 1.1 Hz, 1H), 7.16 (dd, *J* = 11.0, 4.5 Hz, 1H), 6.92 (dd, *J* = 18.6, 10.7 Hz, 2H), 6.76 (d, *J* = 8.0 Hz, 1H)<br><br>**^{13}C NMR (101 MHz, CDCl$_3$):** δ 152.62 (s), 132.58 (s), 129.19 (s), 128.32 (s), 123.18 (s), 121.15 (s), 116.02 (s), 108.49 (s).
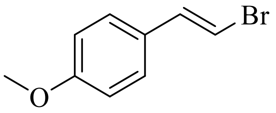<br>1-(2-bromovinyl)-4-methoxybenzene (**3b**)<br>White solid (194.5mg, 61.2% yield)	**^{1}H NMR (400 MHz, CDCl$_3$)**: δ 7.47 (d, *J* = 8.5 Hz, 2H), 7.28 (d, *J* = 13.9 Hz, 1H), 7.11 (t, *J* = 11.3 Hz, 2H), 6.85 (d, *J* = 13.9 Hz, 1H), 4.12 – 3.97 (m, 3H).<br><br>**^{13}C NMR (101 MHz, CDCl$_3$):** δ 159.70 (s), 136.59 (s), 128.81 (s), 127.40 (s), 114.23 (s), 104.04 (s), 55.35 (s).
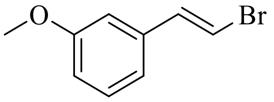<br>1-(2-bromovinyl)-3-methoxybenzene (**4b**)<br>Yellow oil (147.2mg, 46.3% yield)	**^{1}H NMR (400 MHz, CDCl$_3$):** δ 7.31 (d, *J* = 13.9 Hz, 2H), 7.25 (s, 1H), 6.90 (dd, *J* = 16.8, 8.6 Hz, 3H), 3.86 (s, 3H).<br><br>^{13}C NMR (101 MHz, CDCl$_3$) δ 133.04 (s), 129.27 (s), 127.97 (s), 124.77 (s), 120.71 (s), 110.97 (s), 107.89 (s), 99.98 (s), 55.42 (s).
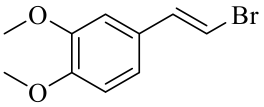<br>4-(2-bromovinyl)-1,2-dimethoxybenzene (**5b**)<br>Yellow brown solid (229.8mg, 63.3% yield)	**^{1}H NMR (400 MHz, CDCl$_3$):** δ 7.06 (d, *J* = 13.9 Hz, 1H), 6.91 – 6.81 (m, 3H), 6.65 (d, *J* = 13.9 Hz, 1H), 3.92 (t, *J* = 5.5 Hz, 6H)<br><br>**^{13}C NMR (101 MHz, CDCl$_3$):** δ 149.22 (d, *J* = 19.6 Hz), 136.79 (s), 129.01 (s), 119.38 (s), 111.16 (s), 108.52 (s), 104.28 (s), 55.89 (d, *J* = 5.4 Hz).
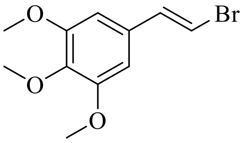<br>5-(2-bromovinyl)-1,2,3 trimethoxy benzene (**6b**)	**^{1}H NMR (400 MHz, CDCl$_3$):** δ 7.42 (d, *J* = 14.0 Hz, 1H), 6.75 – 6.64 (m, 2H), 3.89 (s, 5H), 3.87 (s, 3H).<br><br>**^{13}C NMR (101 MHz, CDCl$_3$):** δ 153.47 (s), 138.41 (s), 137.10 (s), 131.66 (s), 105.88 (s), 103.32 (s), 60.93 (s), 56.17 (s).

Yellow brown oil (153.4mg, 37.6% yield)	
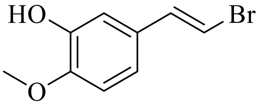<br>5-(2-bromovinyl)-2-methoxyphenol (**7b**)<br>Yellow solid (150.8mg, 44.1% yield)	**^{1}H NMR (400 MHz, CDCl$_3$):** δ 7.06 – 6.85 (m, 2H), 6.84 – 6.71 (m, 2H), 6.60 (d, *J* = 13.9 Hz, 1H), 5.60 (s, 1H), 3.89 (s, 3H).<br><br>**^{13}C NMR (101 MHz, CDCl$_3$):** δ 149.26 (d, *J* = 19.6 Hz), 136.83 (s), 129.06 (s), 119.42 (s), 111.20 (s), 108.56 (s), 104.32 (s), 55.94 (d, *J* = 5.4 Hz).
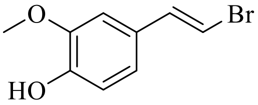<br>4-(2-bromovinyl)-2-methoxyphenol (**8b**)<br>Black solid (181.9mg, 53.2% yield)	**^{1}H NMR (400 MHz, CDCl$_3$):** δ 7.05 – 6.75 (m, 4H), 6.60 (d, *J* = 13.8 Hz, 1H), 5.66 (s, 1H), 3.90 (s, 3H).<br><br>**^{13}C NMR (101 MHz, CDCl$_3$):** δ 145.69 (s), 145.03 (s), 135.94 (s), 127.59 (s), 119.00 (s), 113.63 (s), 107.05 (s), 102.98 (s), 54.95 (s).
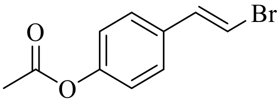<br>4-(2-bromovinyl)-phenyl acetate (**9b**)<br>White solid (104.8mg, 29.1% yield)	**^{1}H NMR (400 MHz, CDCl$_3$):** δ 7.31 (d, *J* = 8.5 Hz, 2H), 7.07 (t, *J* = 11.7 Hz, 3H), 6.73 (d, *J* = 14.0 Hz, 1H), 2.30 (s, 3H).<br><br>**^{13}C NMR (101 MHz, CDCl$_3$):** δ 169.46 (s), 150.66 (s), 136.35 (s), 133.87 (s), 127.27 (s), 122.15 (s), 106.83 (s), 21.29 (s).
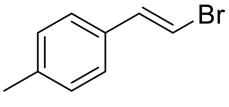<br>1-(2-bromovinyl)-4-methylbenzene (**10b**)<br>Grey white solid (87.6mg, 29.8% yield)	**^{1}H NMR (400 MHz, CDCl$_3$):** δ 7.18 (d, *J* = 7.9 Hz, 2H), 7.12 (d, *J* = 7.9 Hz, 2H), 7.06 (d, *J* = 13.9 Hz, 1H), 6.70 (d, *J* = 14.0 Hz, 1H), 2.32 (s, 3H).<br><br>**^{13}C NMR (101 MHz, CDCl$_3$):** δ 138.27 (s), 137.06 (s), 133.20 (s), 129.50 (s), 126.03 (s), 105.43 (s), 21.29 (s).
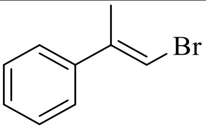<br>1-bromo-2-phenylpropene (**11b**)<br>Brown solid (140.5mg, 47.8% yield)	**^{1}H NMR (400 MHz, CDCl$_3$):** δ 7.39 – 7.22 (m, 5H), 6.44 (d, *J* = 1.0 Hz, 1H), 2.22 (d, *J* = 1.0 Hz, 3H).<br><br>**^{13}C NMR (101 MHz, CDCl$_3$):** δ 141.55 (s), 140.98 (s), 128.56 (s), 127.86 (s), 126.02 (s), 105.41 (s), 19.69 (s).
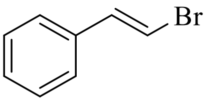<br>2-bromovinyl benzene (**12b**)<br>Yellow oil (82.2mg, 30.1% yield)	**^{1}H NMR (400 MHz, CDCl$_3$)**: δ 7.68 (d, *J* = 7.5 Hz, 1H), 7.40 – 7.30 (m, 3H), 7.27 (t, *J* = 8.0 Hz, 1H), 7.09 (t, *J* = 12.3 Hz, 1H), 6.76 (d, *J* = 14.0 Hz, 1H).<br><br>**^{13}C NMR (101 MHz, CDCl$_3$)**: δ 137.22 (s), 135.97 (s), 132.39 (s), 129.12 – 128.73 (m), 128.47 – 128.18 (m), 126.15 (s), 106.56 (s).

References

1. Yuan, B.; Mahor, D.; Fei, Q.; Wever, R.; Alcalde, M.; Zhang, W.; Hollmann, F., Water-Soluble Anthraquinone Photocatalysts Enable Methanol-Driven Enzymatic Halogenation and Hydroxylation Reactions. *ACS Catal.* **2020,** *10* (15), 8277-8284.
2. Kenta, T.; Mami, K.; Mayumi, S.; Yujiro, H.; Kiyoshi, H., Green-light-driven thioxanthylium-based organophotoredox catalysts: Organophotoredox promoted radical cation Diels-Alder reaction. *Tetrahedron Lett.* **2018,** *59*, 3361-3364.
